# Synthesis of novel azo pyrazole disperse dyes for dyeing and antibacterial finishing of PET fabric under supercritical carbon dioxide

**DOI:** 10.1038/s41598-023-48740-y

**Published:** 2024-01-11

**Authors:** Mamdouh Sofan, Fathy El-Taweel, Adel Abdel-Rahman, Hagar Salman, Elham Negm

**Affiliations:** 1 Chemistry Department, Faculty of Science, Damietta University, New Damietta, Egypt; 2 Chemistry Department, Faculty of Science, Menoufia University, Shebin El-Koam, Egypt

**Keywords:** Chemistry, Materials science

## Abstract

Supercritical carbon dioxide (scCO_2_) has been suggested as a good substitution to environmentally harmful water-based tincturing. The present study describes the successful synthesis of some biologically active dispersion tinctures for supercritical carbon dioxide tincturing of polyester fabric. The coupling of 1-cyanoacetylpiperidine **(1)** with the diazonium salt of aryl amine derivatives (**2a–d)** produced 1-((aryldiazenyl) cyanoacetyl piperidines (**3a–d)**. To create the derivatives of 4-(phenyldiazenyl)-5-(piperidin-1-yl)-1*H*-pyrazol-3-amine **(5a)**, the propane nitriles (**3a–d)** were condensed with hydrazine hydrate. However, the unexpected 3-aminopyrazol-5-ol yellow–red dispersion dyes (**4a–d)** were identified as the reaction results. The MS, IR, and NMR spectra were used to describe the novel dyes, and the results exactly matched the suggested structures. The antibacterial test, which was conducted using the AATCC method, revealed that some of the compounds (**3a–d)** and **(4a–d)** had impressive antibacterial capabilities against the researched +ve and gram −ve bacteria. For eight dyestuffs, the dyeability, color strength, and color fastness of the tincturing process were evaluated. The evaluation focused on determining color uptake using a gauge for color strength (K/S). All dyes displayed excellent rubbing, washing, and light fastness (color change and staining grade of 4–5).

## Introduction

Supercritical tincturing has been proposed as a good substitute for environmentally harmful water-based dyeing because it uses supercritical carbon dioxide (scCO_2_) rather than water as the dyeing solvent, and it doesn't need the addition of chemicals like dispersants and surfactants. The scCO_2_, which has a high density, a solvating power comparable to liquid solvents, a low viscosity, and diffusivities like supercritical gases can be easily adjusted to have a high solvating power by altering the sys’s pressure and temperature. The tincturing of polyester and other synthetic fabrics in scCO_2_ solvent can be attributed to its ability to dissolve dispersed tincturing as well as its swelling and plasticizing effects on hydrophobic polymers^1–4^. In addition to our previous research to develop the disperse dyes and dyed polyester fabrics in scCO_2_^5–10^, we have made an effort to offer a broad color spectrum of dyed polyester materials that are both antibacterial and colorfast. The first stage was the preparation of novel aminopyrazole dyes with good antibacterial activity. Textiles manufactured of polyester were eco-dyed using a dispersed dye in scCO_2_, and its dyeing ability, hue, and fastness were evaluated^11,12^.

Heterocyclic amines have been widely used in the preparation of disperse dyes due to their excellent affinity to discharge on polyester fibers^13,14^. The biological activity of the heterocyclic dyes containing piperidine and pyrazole moieties was also studied. Piperidine is an organic heterocyclic amine that is commonly employed as a building block and reagent in the production of chemical compounds, including medicinal products. Piperidine-containing compounds have a wide range of biological functions and are used as antibacterial, anti-inflammatory, and antipsychotic medicines in a variety of ways^15,16^.

The current work aims to investigate the synthesis of aminopyrazole derivatives using widely accessible starting materials and their application to polyester fabrics under scCO_2_. To decide the dye effectively for the fabrics, several fastness tests including washing, light, and rubbing were also investigated.

## Experimental sections

### Materials and characterization

The reactant materials were gotten from Sigma Aldrich, and the polyester textiles were purchased from Misr for Spinning and Weaving Company in Mahalla El-Kobra, Egypt. All uncorrected melting points were determined in degrees Celsius, using a Griffin melting point equipment in open capillary tubes. The IR spectra of KBr pellets were measured by using a JASCO 410 spectrometer, and just a few absorptions in the 4000–400 cm^−1^ range were detected. ^1^H and ^13^C NMR spectra were identified using a Bruker Avance (III 400 MHz and a Joel ECA-II 500 MHz) NMR spectrometer using CDCl_3_ and DMSO-d_6_ as the solvent. Chemical shifts (δ) are calculated in ppm downfield and present of tetramethyl silane (TMS) as an internal reference. A Kratos 70 eV MS system or a Varian MAT 311A Spectrometer were both used to record the mass spectra (MS). Analytical thin-layer chromatography (TLC) was carried out on silica gel G60 F (Merck) plates that were precoated to a thickness of 0.2 mm. TLC was observed using iodine vapour or UV light (254 and 366 nm). The supplemental information provided Raman spectra, CIELAB colour space, and chemical analyses of generated tinctures (^1^H NMR, ^13^C NMR, MS, and FTIR spectra).

## Chemical analysis and tincture synthesis

### Dye synthesis

The synthesis process for the intended dispersed dyes included, firstly, the preparation of 2-arylazo-3-oxopropanenitrile derivatives **3a–d** by coupling diazonium salt of aryl amine derivatives **2a–d** with 1-cyanoacetyl piperidine **(1)** at 0 °C. Subsequently, a condensation reaction on compound **3a–d** with hydrazine hydrate was performed in absolute ethanol to afford the products (**4a–d)** in excellent yields.

### Preparation method of 1-((aryldiazenyl) cyanoacetyl) piperidine (3a–d)

Aryl amine **2a–d** (5 mmol) was deliquesced in water (7 mL) containing conc. HCl (5 mL). The flipping arylamine hydrochloride solution was cooled to (0–5) °C, and dropwise additions of NaNO_2_ (6 mmol) in water (3 mL) were then added while the reaction’s temperature was kept below 5 °C. The solution of diazonium chloride was flipped for 30 min at 5 °C and then gradually added to the solution of 1-cyanoacetyl piperidine **(1)** (5 mmol) in 20 mL of ethanol combined with a solution of sodium ethanoate (3 g) in 5 mL of water. After being flipped for 60 min at (0–5) °C, the coupling reaction contents were swirled for an hour at normal temperature. The precipitate was nominated and submerged in cold water many times. The products **3a–d** were dried, purified in ethanol, and then used in the subsequent step.

#### Characterization of 3-oxo-2-(phenyl diazenyl)-3-(piperidin-1-yl) propane nitrile (3a)

A brown crystal from ethanol, 85% yield, m. p. (114–116) ^o^C, IR (*ν*/cm^−1^): 1473–1439 (N=N), 1554(C=C_aromatic_), 1605 (C=O), 2197 (CN), 3454 (NH); ^1^H NMR: (400 MHz, chloroform-D): δ 1.754 (6H, 3CH_2_, q), 3.775 (4H, d, (CH_2_)_2_N), 7.146–7.175 (1H, Ar–H, t, J = 7.5 Hz), 7.242–7.26 (2H, Ar–H, d, J = 9 Hz); 7.39–7.419 (2H, Ar–H,t, J = 7.5 Hz), 9.228 (1H_exchange with D2O_, NH, S); MS (ESI) m/z Calc. for C_14_H_16_N_4_O (256.13). Found 256.94 [M^+^].

#### Characterization of 2-((4-methoxyphenyl) diazenyl)-3-oxo-3-(piperidin-1-yl) propanenitrile (3b)

A brown crystals from ethanol, 86% yield, m. p. (116–118) ^o^C, IR (ν/cm^−1^): 1482 (N=N), 1543 (C=C_aromatic_), 1609 (C=O), 2203 (CN), 3408 (NH); ^1^H NMR: (400 MHz, chloroform-D): δ 1.69 (6H, 3CH_2_ ,q), 3.7 (4H, (CH_2_)_2_N, t), 3.75 (3H,${\text{O}}({\text{CH}}_{3} ),{\text{ s}}$), 6.9–6.92 (2H, Ar–H, d, J = 8 Hz), 7.17–7.19 (2H, Ar–H, d, J = 8 Hz), 9.3 (1H, NH_exchange with D2O_, s)). ^13^C-NMR (100.63 MHz, CDCl_3_): δ 24.31, 25.85, 55.59, 55.63, 108.67, 110.94, 114.78, 116.28, 134.69, 156.89, 160.00; MS (ESI) m/z Calc. for C_15_H_18_N_4_O_2_ (286.34). found 286.17 [M^+^].

#### Characterization of 2-((4-methoxy-2-nitrophenyl) diazenyl)-3-oxo-3-(piperidin-1-yl) propane nitrile (3c)

A brown crystals from ethanol, 84% yield, m. p. (108–110) ^o^C; IR (ν/cm^−1^): 1434 (N=N), 1515 (C=C_aromatic_), 1630 (C=O), 2205 (CN), 3424 (NH); ^1^H NMR: (400 MHz, chloroform-D): δ 1.74 (6H,3CH_2_, q), 3.73 (4H, (CH_2_)_2_N, t), 3.9 ( 3H,${\text{ O}}({\text{CH}}_{3} ),{\text{ s}}),{ }$ 7.28–7.31 (1H, Ar–H, m, J = 8 Hz), 7.67–7.73 (1H, Ar–H, m, J = 4 Hz), 7.93–7.96 (1H, Ar–H, d, J = 8 Hz), 11.82(1H, NH_exchange with D2O_, S); ^13^C-NMR (100.63 MHz, CDCl_3_): δ 24.24, 25.53, 48.24, 56.05, 107.69, 110.14, 114.15, 118.32, 124.77, 132.16, 134.21, 155.15, 159.04.

#### Characterization of 2-((2-chlorophenyl) diazenyl)-3-oxo-3-(piperidine-1-yl) propane nitrile (3d)

An orange crystals form ethanol, 89% yield, m. p. (148–150)^o^C; IR (ν/cm^−1^) 1459 (N=N), 1498 (C=C_aromatic_), 1623 (C=O), 2212 (CN), 3417 (NH); ^1^H NMR: (400 MHz, chloroform-D): δ 1.75 (6H, 3CH_2_, q), 3.7–3.76 (4H, (CH_2_)_2_N, t), 7.18–7.22 (1H, Ar–H, m, J = 8 Hz), 7.67–7.71 (1H, Ar–H, m, J = 8 Hz), 7.78–7.8 (1H, Ar–H, d, J = 8 Hz), 8.27–8.3 (1H, Ar–H, m, J = 8 Hz), 11.95(1H_NH exchange with D2O_, S); ^13^C-NMR (100.63 MHz, CDCl_3_): δ 24.39, 25.54, 44.48, 109.9, 115.44, 116.87, 122.93, 125.93, 134.05, 136.06, 138.02, 158.78.

### Preparation method of 3-amino-4-(aryl diazenyl)-1*H*-pyrazole-5-ols (4a–d)

Refluxing was performed for 6 h using a combination of compound **3a–d** (10 mmol) and hydrazinium hydroxide (10 mmol) in anhydrous ethyl alcohol (25 ml). The precipitate obtained after the mixture warmed to room temperature was nominated and recrystallized from the ethanol to produce compound **4a–d**.

#### Characterization of 3-amino-4-(phenyl diazenyl)-1*H*-pyrazol-5-ol (4a)

A wine red from ethanol, % 65 yields, m. p. > 250 °C; IR (ν/cm^−1^): 1482 (N=N), 1641 (C=C_aromatic_), 3275–3250 (NH_2_), 3200 (NH), 3376 (OH); ^1^H NMR (400 MHz, DMSO-d_6_): δ 5.85 (2H, NH_2 exchange with D2O_, s), 7.101–7.116 (1H, Ar–H, t, J = 8 Hz), 7.355–7.415 (2H, Ar–H, t, J = 8 Hz), 7.512–7.556 (2H, Ar–H, d, J = 7 Hz), 10.548 (1H, OH_exchange with D2O_, s), 12.911 (1H, NH_exchange with D2O_, S); MS (ESI): m/z Calc. for C_9_H_9_N_5_O (203.21). found 203.89 [M^+^].

#### Characterization of 3-amino-4-((4-methoxyphenyl) diazenyl)-1*H*-pyrazol-5-ol (4b)

A dark brown crystals from ethanol, 70% yield, m. p. (252–254) ^o^C; IR (ν/cm^−1^): 1498 (N=N), 1641 (C=C_aromatic_), 3341–3209 (NH_2_), 3433 (OH); ^1^H NMR (500 MHz, DMSO-d_6_): δ 3.75(3H, ${\text{O}}({\text{CH}}_{3} ),{\text{ s}}$), 5.85 (2H, NH_2 exchange with D2O_, s), 6.97–6.99 (2H, Ar–H, d, J = 8 Hz), 7.49–7.52 (2H, Ar–H, d, J = 8 Hz), 10.49 (H, OH_exchange with D2O_, s), 13.01 (H_exchange with D2O_, NH, s); ^13^C-NMR (100.63 MHz, DMSO): δ 55.84, 115.15, 117.01,122.58,135.89. 150.45, 156.94, 159.34; MS (ESI) m/z Calc. for C_10_H_11_N_5_O_2_ (233.23). Found 234.44 [M^+^].

#### Characterization of 3-amino-4-((4-methoxy-2-nitrophenyl) diazenyl)-1*H*-pyrazol-5-ol (4c)

A brown red crystals from ethanol, 66% yield, m. p. > 250 °C; IR (ν/cm^−1^): 1422 (N=N), 1621 (C=C_aromatic_), 3075 (CH unsaturated), 3370–3266 (NH_2_), 3465 (OH); ^1^H NMR (500 MHz, CDCl_3_): δ 3.02 (3H, OCH_3_, s), 6.1 (2H_exchange with D2O_, NH_2_, S), 7.52 (1H, Ar–H, m, J = 8 Hz), 7.67 (1H, Ar–H, m, J = 4 Hz), 8.25 (1H, Ar–H, m, J = 8 Hz), 10.71 (1H_exchange with D2O_, OH, S), 14.21 (1H_exchange with D2O_, NH, S); ^13^C-NMR (100.63 MHz, DMSO) δ 56.5, 108.04, 118.68, 125.33, 128.09, 133.25, 134.64, 150.03, 155.15, 158.32.

#### Characterization of 3-amino-4-((2-chlorophenyl) diazenyl)-1*H*-pyrazol-5-ol (4d)

A brown crystals from ethanol, 70% yield, m. p. > 250 °C; IR (*ν*/cm^−1^): 1498 (N=N), 1616 (C=C_aromatic_), 3377–3236 (NH_2_), 3477 (OH); ^1^H NMR (500 MHz, CDCl_3_): δ 6.17 (2H_exchange with D2O_, NH_2_, d), 7.3 (1H, Ar–H, m, J = 8 Hz), 7.84 (1H, Ar–H, m, J = 8 Hz), 8.26 (1H, Ar–H, m, J = 8 Hz), 8.31 (1H, Ar–H, m, J = 8 Hz), 10.77 (1H_exchange with D2O_, OH exchange with D_2_O, s), 14.25 (1H_exchange with D2O_, NH, S); ^13^C-NMR (100.63 MHz, DMSO) δ 117.01, 123.48, 126.26, 129.35, 134.42, 136.75, 139.08, 149.98, 158.13.

### Dyeing polyester fabrics under the scCO_2_ medium

The polyester fabric must first be prepared for coloring (5 × 5 cm) (0.5 g). It was lapped around a perforated beam and then suspended inside the 50 ml interior tincturing stainless steel receptacle. The bottom of the dyestuff receptacle was filled with the purified dyestuff (2, 3, and 4%) (By the investigated concentration).

CO_2_ previously stocked in a gas canister was cooled to the point where it turned into a liquid using a high-pressure regulator (model JASCO BP-4340) and a CO_2_ pressure pump (type JASCO PU-4386). After the carbon dioxide distribute system was turned on, the tincturing process began. The proper temperatures (110, 120, 130° C) and pressures (15, 20, 25 MPa) were set. Throughout the tincturing process, the heat-restitution jacket (type HC-2068-01) maintained a steady temperature.

The scCO_2_ and the tinctured polyester fabrics were adjudicated in the separator unit. The temperature was gradually lowered, and the pressure was reduced to atmospheric pressure when the predetermined time had elapsed. The tinctured polyester samples were then eluted from the tincturing receptacle for extra realization. Polyester fabric samples were laundered in acetone at room temperature to elute unfixed colors that had adhered to the surface. After that, the samples were cleaned for 20 min at 70 °C with a 2% nonionic detergent (Sera Fast CRD) to evaluate wash fastness and laundering durability (up to 10 washing cycles).

## Testing and measurement analysis

### Color evaluation

A spectrophotometer was used to perform the measurements of the lightness (L*), chroma (c*), hue (h*), degree of redness (+ve) and greenness (−ve) (a*), and degree of yellow (+ve) and blueness (−ve) (b*).

The measurements of the lightness (L*), chroma (c*), hue (h*), degree of redness (+ve) and greenness (−ve) (a*), and degree of yellow (+ve) and blueness (−ve) (b*) were performed by using a spectrophotometer. The color strength (K/S) of the tinctured samples and the associated CIELAB coordinates express the tincture uptake (model CM-3600A, manufacturer KONICA MINOLTA, Japan). The AATCC standard techniques, such as (AATCC-61-2A-1996) for washing^17^, (AATCC 8-2001) for rubbing^18^, and (AATCC-16A-1972) for light fastness^19^ were used to assess the color fastness of colored polyester textiles under optimal parameters. The stability and resistance of the new tinctures on the polyester surface during repeated washing cycles (up to 10 washing cycles) were evaluated by using AATCC Test Method 61 (2A)-1996^20^. According to test method (AATCC 15-1997), dyed polyester fabrics were submitted to sweating fastness testing^18^.

To determine the dye fixation and to remove the excess dye molecules that were unfixed to the substrate during the dyeing procedure, a half piece of the dyed cotton was extracted in a Soxhlet extractor for 30 min with a 50 wt% solution of acetone in water. The dye fixation was calculated based on the degree of adsorbed dye on the cotton (Eq. 1) after dyeing and after being extracted.

1 $$ {\text{F (Fixation}}\;\% {) } = {\text{K}}/{\text{S (afte extraction) }}/{\text{ K}}/{\text{S (after dyeing) }} \times 100\% $$

### Raman spectra of polyester samples, both dyed and undyed

Using the Jasco NRS-4500, Raman spectra between 200 and 4000 cm^−1^ were obtained.

### Microbiological resistance of the new dyes

From Agric, four different microbial species were kindly obtained. These microorganisms included *Escherichia coli, Pseudomonas aeruginosa, Bacillus cereus, and Staphylococcus aureus* ATCC (25922, 27853, 6633, and 25923) respectively. Until utilization, the bacterial strains were kept on NA medium slants at 5 °C. The microbial strains were sub-cultured on fresh NA media slants and incubated at 37 °C for 2 days before to use. Each slant received 5 mL of sterile saline solution (0.09% NaCl). A sterile inoculating loop was applied to gently brush the bacteria's cells loose. To completely remove all bacterial cells from slants, a vortex mixer was used for 1 min. Applying well diffusion techniques, the antibacterial activity was assessed in 20 mL of nutrient agar media in the form of petri plates. Using a sterile cotton swab, the suitable microbial strains were put onto each plate. Four tiny wells measuring 6.3 mm in diameter were then created by a sterilized cork borer. After 1 day, the dyes inhibition zones were meticulously measured^21,22^.

## Results and discussion

### Discussion of the synthetic strategy

Utilizing a green technique with a high yield without using solvent was synthesized 1-cyanoacetyl piperidine **(1)** that coupled with diazonium salt of aryl amine derivatives (**2a–d)** to yield 3-oxo-2-(phenyl diazenyl)-3-(piperidin-1-yl) propanenitrile (**3a–d).** Refluxing the compounds (**3a–d)** with hydrazine hydrate in ethanol afforded the unexpected 5-amino-4-arylazopyrazole derivatives (**4a–d)** in a novel procedure^23^. The structures of the tinctures were created on basis of their both correct elemental analysis and spectral data. Thus, the FT-IR method was used to confirm the functional group of the dyes, in sample **3a** the presence of bands at 3454.8 cm^−1^ was attributable to NH groups, the strong absorption bands at 2197 and 1630.5 cm^−1^ are assigned for the characteristic of CN and carbamate CO stretching vibrations respectively and the absorption bands at 2937.06 cm^−1^ to the stretching vibrations of the CH saturated group. The ^1^H-NMR disclosed the presence of signals at δ 1.754 and 3.77 ppm characteristic of piperidine ring and signal at δ 9.228 ppm characteristic of NH exchangeable with D_2_O indicating to its existence in hydrazo form. IR spectra of (**4a–d)** showed the presence of absorption peaks at 3376.7, 3250.3, and 3275.5 cm^−1^ attributable to OH, and NH_2_ groups. In addition to aromatic protons, H-NMR spectra of (**4a–d)** exhibit signals at δ 12.911, 10.548, and 5.85 ppm characteristic for NH, OH, and NH_2_ exchangeable with D_2_O, respectively.

### Proposed mechanism for disperse dyes

In contrast to expectations, the ring closure of compounds (**3a–d)** produced the corresponding 3-amino-5-hydroxy-4-arylazo pyrazole derivatives (**4a–d)**, not 5-piperidin-1-yl derivatives (**5a–d)** in reaction with hydrazine hydrate. ^1^H NMR showed the disappearance of the proton signals attributable to piperidine ring at δ 1.754—3.77 ppm. The **FT-IR** indicated to the disappearance of the most important characteristic bands of carbonyl and cyano groups (1630 and 2197 cm^−1^). The reaction may be proceeded via the nucleophilic addition of H_2_NNH_2_ to C=O of the tertiary amide followed by elimination of piperidine.

### Color characteristics

**Figure 1:**
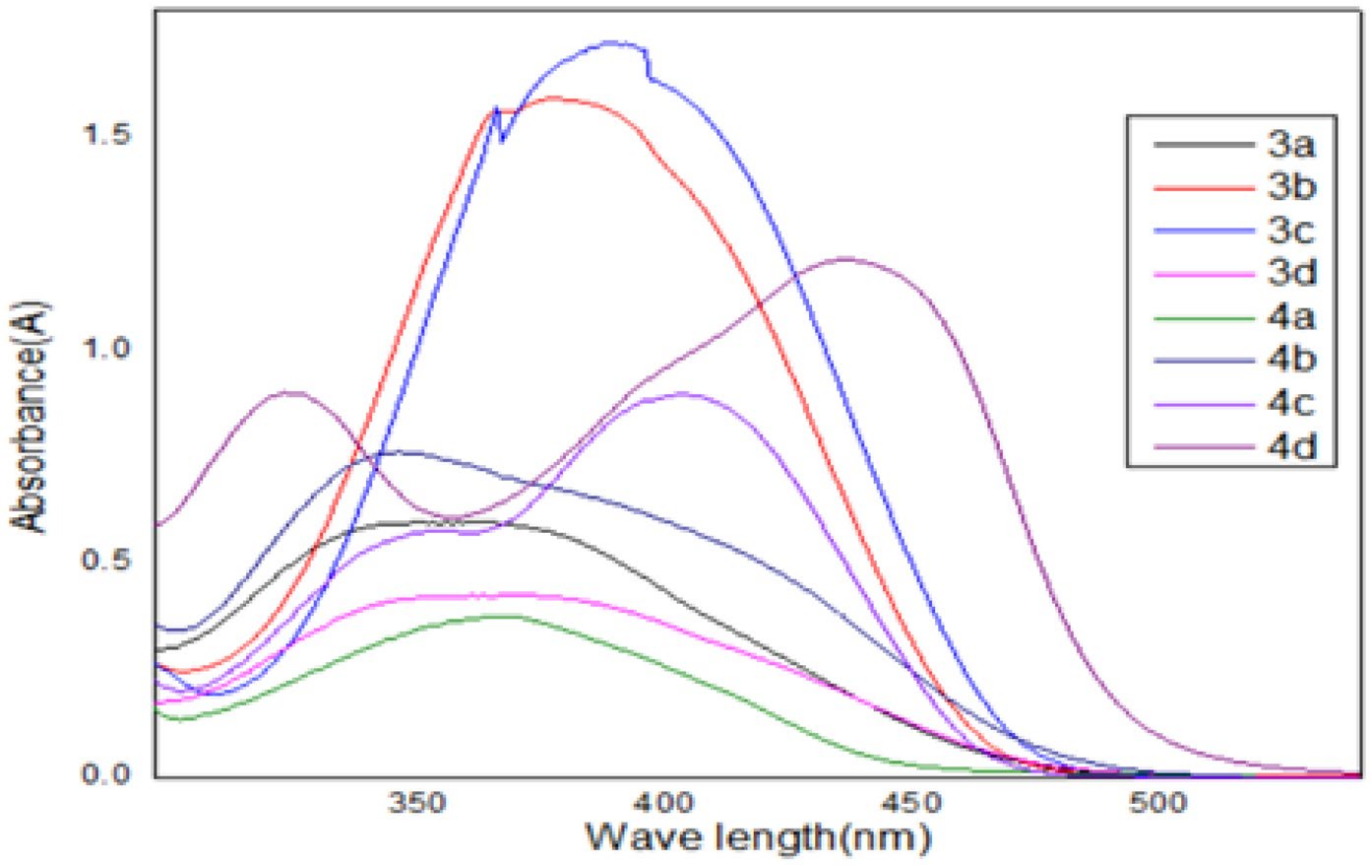
Spectra of dyestuff in chloroform in the visible and UV spectrum (the λ_max_ (nm) = 352, 385, 395, 372, 380, 345, 446, and 330–421 for 3a, 3b, 3c, 3d, 4a, 4b, 4c, and 4d, respectively).

The UV–visible absorption spectra of synthetic disperse dyes were obtained by utilizing 10^–4^ L (mol cm)^−1^ of tincture concentrations in methylene chloride (nonpolar) solvent. (Fig. 1 and Table 1) depicts the results. The electronic spectra of **all dyes** showed λ_max_ in the region of (330–450) nm in CH_2_Cl_2_. These bands were produced by the entire conjugated system's electronic transitions, including the diazene group characterized as the π–π* form transition, heterocyclic moieties, and phenyl rings. A significant bathochromic change (blue shift) was produced by the extension of the electrons caused by π–π* in a chromophoric system with drawing groups.

**Table 1:** The dispersed dyes absorption of ultraviolet light.

Sample	λ_max_ (nm)	The molar extinction coefficients (ε M^−1^ cm^−1^)*10^3^
3a	352	6.2
3b	385	16
3c	395	17.3
3d	372	4.4
4a	380	4
4b	345	7.2
4c	446	11.3
4d	330–421	9–12.1

It's widely recognized that synthetic dyes have excellent visual light absorption properties and exhibit brilliant red to orange colors in non-polar solvents. Moreover, all the synthetics disperse dyes display suitable dissolutions in non-polar solvents. This may confirm the effectiveness of utilizing these dyes in non-polar supercritical carbon dioxide tincturing.

### Factor affecting the ability of polyester fabrics to acquire dye

**Figure 2:**
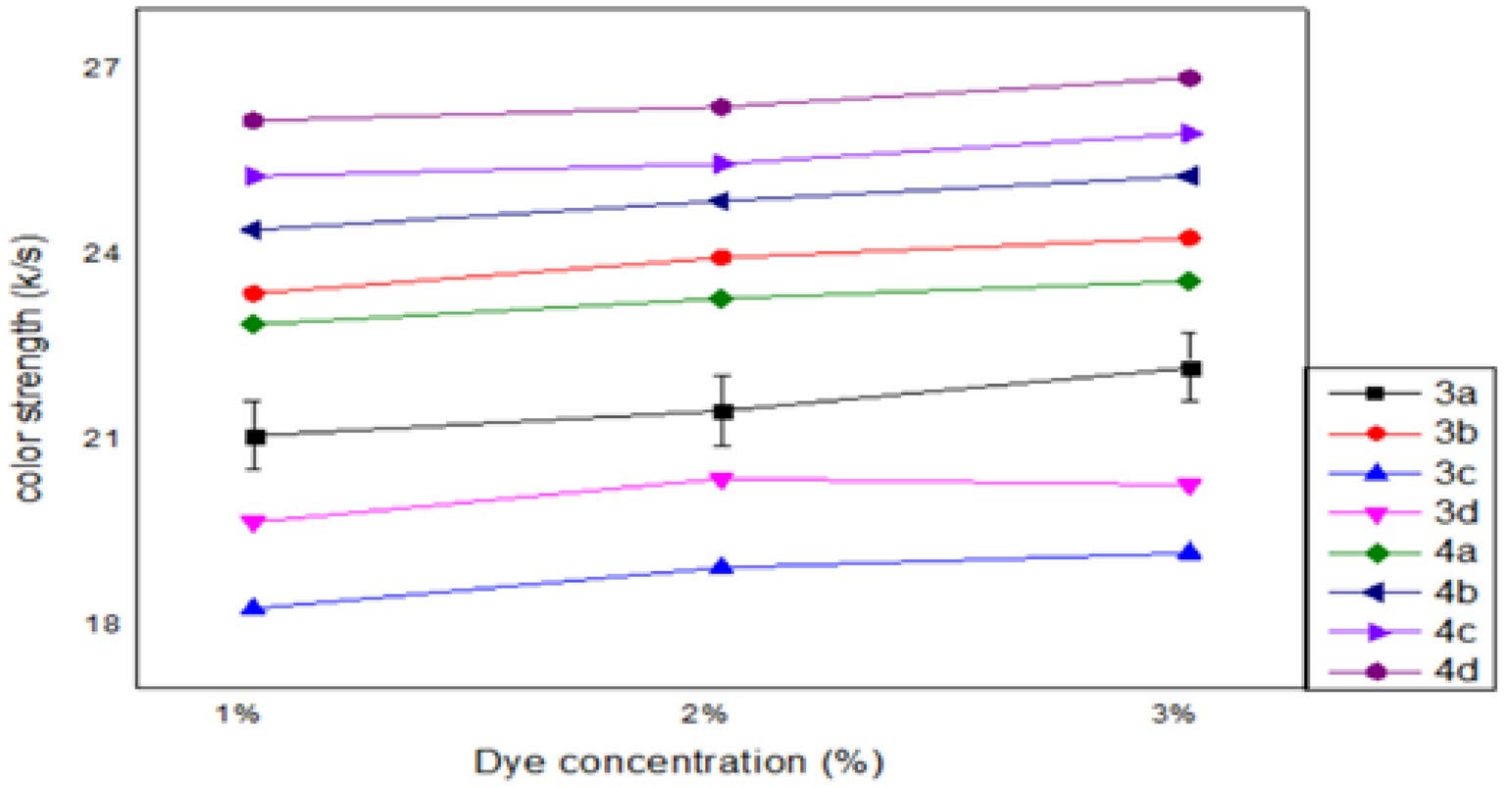
Effect of dyed concentration (1, 2, 3%) on dyed polyester fabrics. The optimum concentration is 3% for all dyed except 3d is 2%.

**Figure 3:**
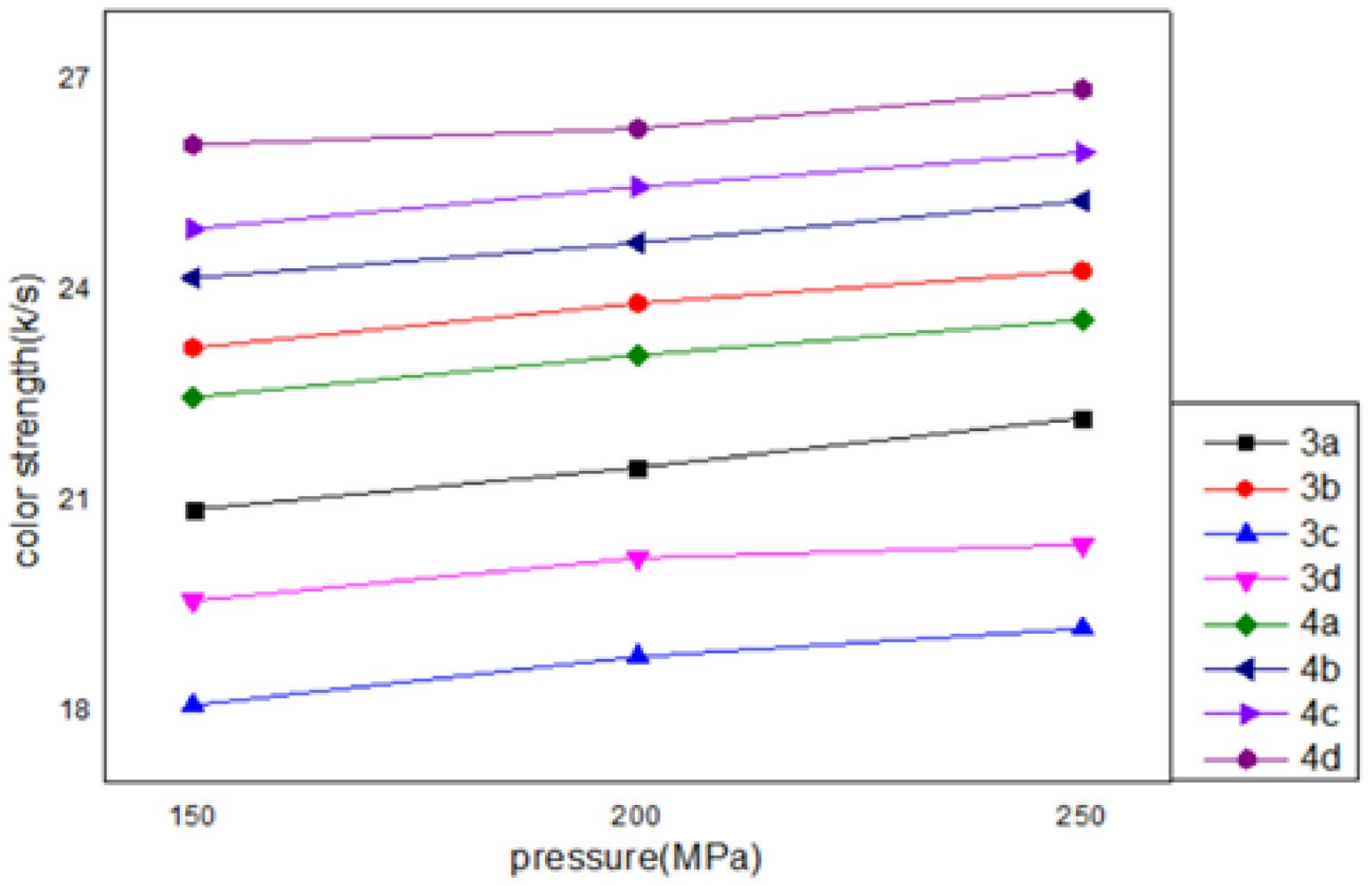
Effect of pressure (15, 20, 25 MPa) on dyed polyester fabrics. The optimum pressure for all dyes is 25 MPa.

**Figure 4:**
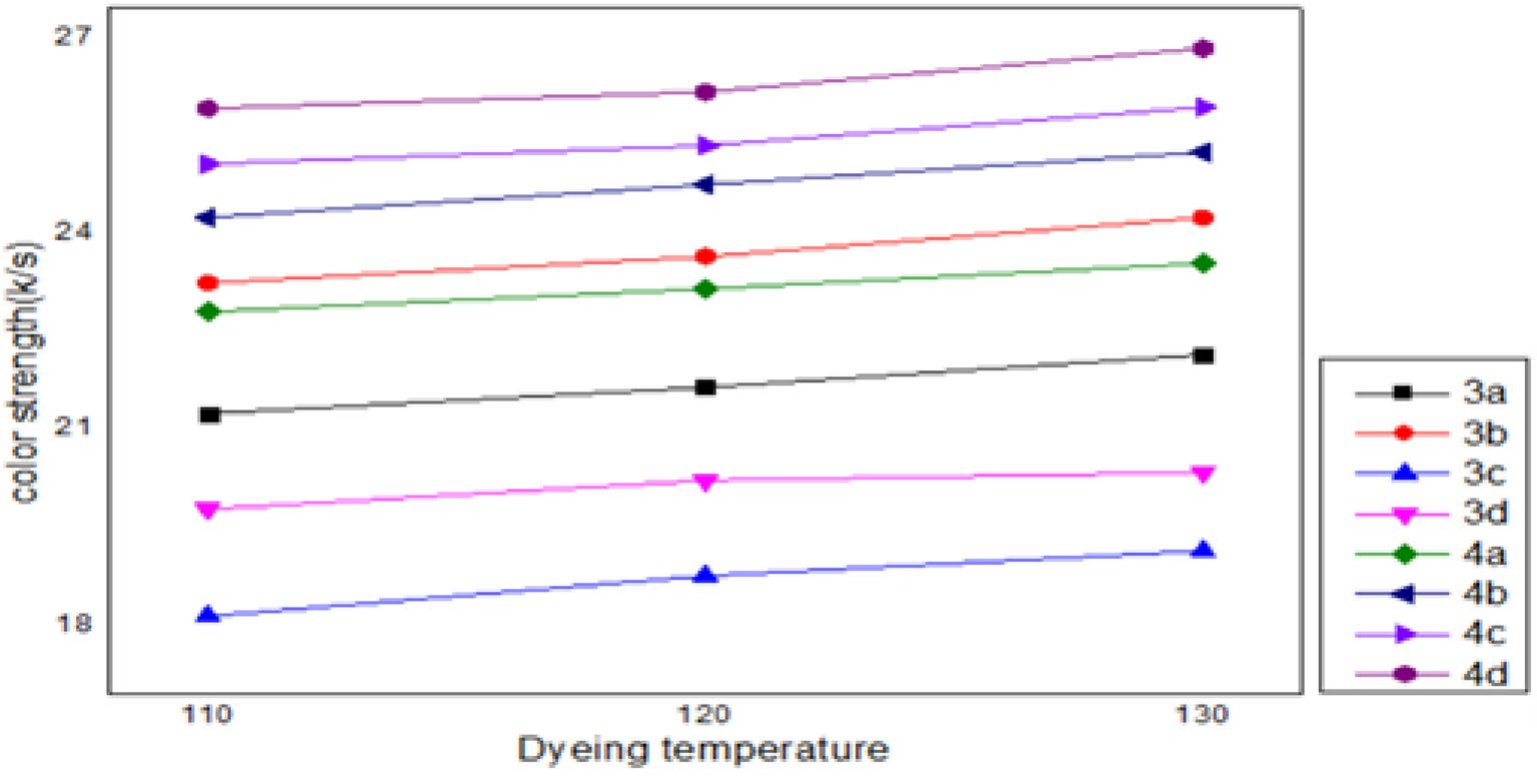
Effect of temperature (110, 120, 130 °C) on dyed polyester fabrics. The optimum temperature for all dyes is 130 °C.

**Figure 5:**
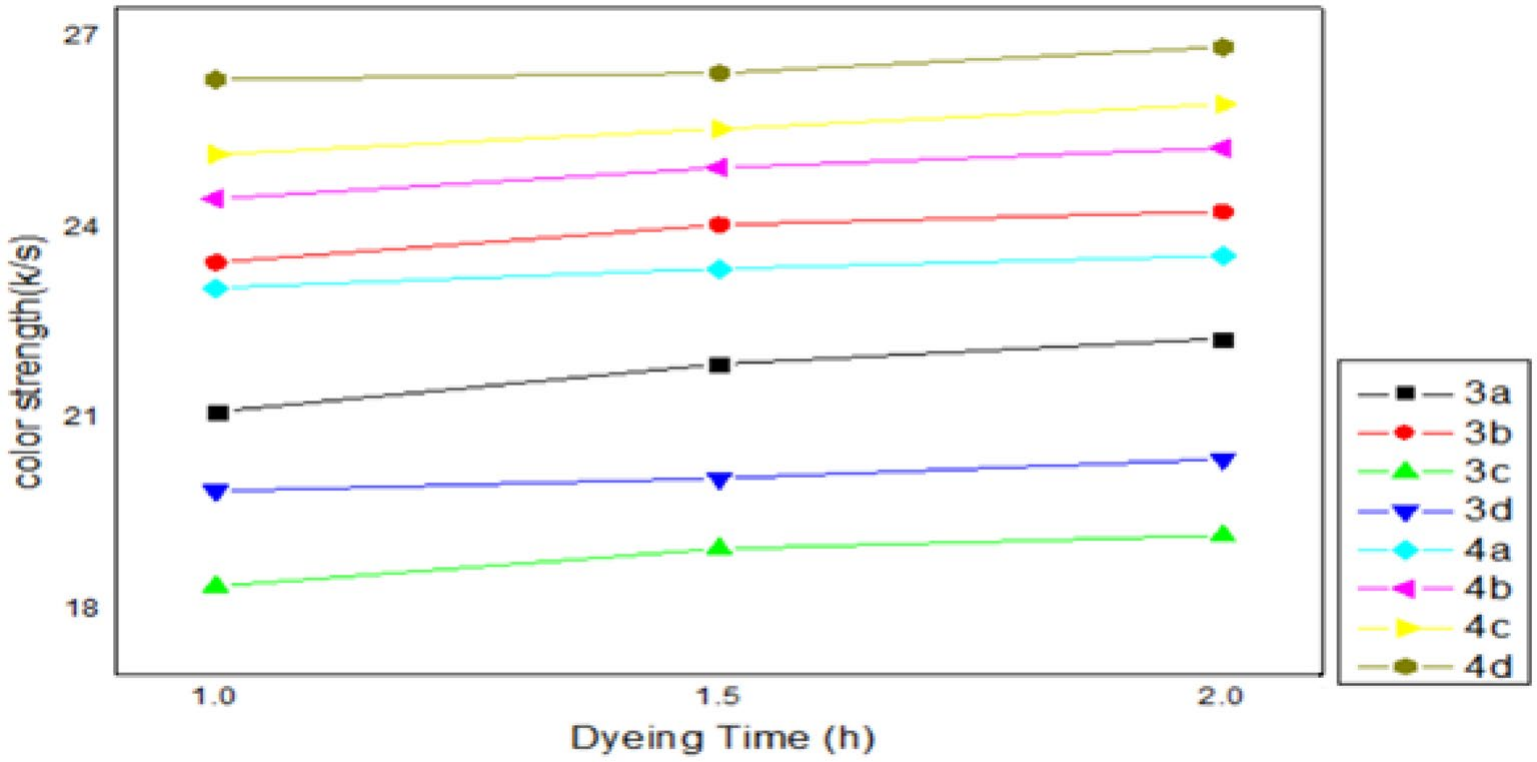
Effect of dyeing time (60, 90, 120 min) on dyed polyester fabrics. The optimum dyeing time for all dyes is 120 min.

The optimum conditions of the dye’s ability to dye the polyester could be determined through conducting experiments that demonstrate the effect of the most crucial factors, such as temperature (110, 120, 130 °C), pressure (150, 200, 250 MPa), dye concentration (1, 2, 3%) and dyeing time (60, 90, 120 min), on the dyeing process. The impacts of tincture concentration, pressure, temperature, and time factors on the color strength of polyester fabrics were designed in the following Figs. 2, 3, 4, 5.

The color strength *(K/S)* of the polyester fabric that was tinctured in scCO_2_ medium is affected by the tincture concentration at a fixed system temperature of 130 °C, a pressure of 25 MPa, and a tincturing time of 60 min. According to Fig. 2, the values of *K/S* of the tinctured polyester samples increased linearly from 1 to 3% for all tinctures except for **3d**. The maximum adsorption, per the findings, betided at a tincture concentration of 3%. This outcome was attained as a result of the tincture molecules in scCO_2_ being preferentially adsorbed and forming a surface layer on the fiber surface. As the tincturing process proceeds, the tincture molecules gradually migrate from the top layer into the amorphous regions of the polyester fibers. With increasing tincture concentration from 1 to 3% in the case of the tincture **3d**, the color strength first increased and subsequently declined. The significant saturation of tincture at low concentrations can be blamed for the subsequent decline at 3%.

With a system pressure (15–25 MPa), a temperature (130 °C), and a tincturing time (1 h), the effects of the system pressure on the color strength of (2%) tinctured polyester were investigated. As shown in Fig. 3, the K/S of the tinctured polyester textiles increased with increasing system pressures in the range of 15 to 25 MPa. As is common knowledge, pressure plays a critical role in the scCO_2_-tincturing process of fabrics. Improved color strength values resulted from the gradual rise in tincture solubility and CO_2_ fluid density at constant temperature and pressure. Furthermore, because of the growing interaction between the hydrophobic medium and the intermolecular chains of the textiles, the soluble tincture molecules may spread very rapidly into the amorphous region of the inflated fibers. The tincture molecule might thus be absorbed into the polyester fibers more successfully.

The dye's solubility may alter as a result of changes in temperature. As a result, it was determined that the tincture absorbency and tincturing temp (100, 120, and 130 °C) in scCO_2_ correlated, as depicted in Fig. 4. A low tincture percentage of 2%, a fixed 25 MPa, a 1 h tincturing period, and an average tincture medium flow rate of 9.500 ml/min were called in the experiment. It was found that as system temp rose from 100 to 130 °C, the color strength of the polyester cloth tinctured in scCO_2_ grew stronger. This result can be explained by the fact that supercritical carbon dioxide (scCO_2_) has a higher density as dyeing temperatures rise, the dyes are more soluble, and the dye concentration is dispersed more evenly throughout the interior.

According to Fig. 5, polyester fabric was tinctured at 130 °C and 25 MPa for durations of 60 to 120 min. The K/S values on the tincture polyester samples up trended steadily until the dyeing time was increased to 120 min, at which point they peaked. This outcome might have occurred because it took a comparable amount of time for the tincture to migrate from the fiber's surface to its Centre.

The results indicate that the ideal system parameters of 130 °C, 25 MPa, 120 min of tincturing, and 3% of tincture concentration were achieved for dying polyester textiles with tinctures in scCO_2_. The value of the k/s of the dyes 3a, 3b, 3d, 4a, 4b, and 4d is higher than 3c and 4c due to the presence of the donating group (H, OCH3 and CL) which causes the depth of color.

### Color fastness test

The perfect samples achievable were utilized for all experiments. The ideal tincturing parameters in scCO_2_ were 3% tincture concentration, 130 °C for the tincturing temperature, 120 min for the tincturing time, and 25 MPa for the tincturing pressure. Efficiency of tincturing with K/S is significantly influenced by colorfastness. The results of washing, rubbing, and light fastness tests showed that all the tinctured polyester fabrics (**3a–d),** (**4a–d)** in scCO_2_ medium gave excellent fastness values according to the geometric grey scale. All of the tinctured polyester samples demonstrated outstanding rubbing (both wet and dry), scoring a grade of 5, according to the results of the washing fastness test (rating between 4 and 5). The light fastness of all dyes yielded very good to exceptional results with grades of 4, 4-5, and 5. New dyes' stability, exchangeability, and durability on the polyester surface against repeated washing cycles (up to 10 washing cycles) were assessed using AATC Test Method 61(2A)-1996. The perspiration fastness test against acid and alkali to fading and staining of tincturing polyester fabric was examined by using (AATCC 15-1997) test method. According to the results' global grayscale, all the tinctures displayed outstanding grades. In acidic media, the fading results reached 4–5 to 5 grades for all tinctures. All the tinctures achieved a staining grade of 4–5. Similarly, in alkaline media, all the tinctures exhibited excellent grades on fading and staining tests. The test results were summarized in Table 2.

**Table 2:** Fastness characteristics of polyester fabrics dyed (values between parentheses are the durability of wash fastness) (CF, color fastness) (units from 0 to 5: 0 is the lowest and 5 is the highest).

Test standards		3a	3b	3c	3d	4a	4b	4c	4d
CF to crocking (AATCC 8)	Dry	5	5	5	5	5	5	5	5
	Wet	4–5	4–5	4	4–5	4–5	4–5	4–5	4–5
CF to washing (AATCC 61-2A)	Color change	4–5	4–5	4	4	4–5	4–5	4–5	4–5
	Staining	5	4–5	4–5	4–5	5	5	5	5
CF to perspiration (AATCC 15)	Alkaline	Fading	5	4–5	5	5	5	4–5	5
		Cotton	5	5	5	5	5	4–5	4–5
		Polyester	5	5	4–5	4–5	5	5	5
	Acidic	Fading	4–5	5	5	5	4–5	5	5
		Cotton	5	4–5	5	4–5	5	5	5
		Polyester	5	5	4–5	4–5	5	5	5
CF to light (AATCC 16A-1972)			4	4–5	5	4–5	5	4–5	5

### Color assessment

We assessed the color coordinates of the dyed polyester fabrics using CIELAB theory and represented them in terms of L* a*, b*, C*, and h (the data are shown in Table 3). The number on the L* axis, which represents "lightness," rises from 0 to 100 as the lightness shifts from black to white. To demonstrate how the color shifts from red to green in the color space, the indicators of a* go from positive to negative. The value of b* shifting from positive to negative represents the color shifts in the color space from yellow to blue. Chroma denotes brightness, C* denotes chroma, and h denotes the hue angle within the range of 0° to 360° in the color space. The findings showed a strong affinity between the dyes and polyester materials, which frequently result in vivid and deep hues ranging from yellow to orange. According to the positive values of b*, the color hues of all synthetic dyes on polyester fabric are moved towards the yellowish direction of the yellow-blue axis. The new dyes' color hues on the polyester fabric are shifted in a reddish manner along the red-green axis based on the positive value of a*. ∆E value represents the overall color difference of the dyed sample, with larger values indicating a greater difference from the color of the undyed fabric, and smaller values indicating a smaller difference. Eight dispersed dyes displayed brighter and duller colors, respectively, in accordance with the color brightness (C*) values. At the saturation point, deep colours were obtained with the amino pyrazole dye for PET fabrics, showing the largest fixation of the series percentages between 89.58% and 96.01%, as shown in Table 4.

**Table 3:** Color coordinates and K/S of dyed polyester fabric in scCO_2_. (L*) Stand for lightness, (a*) for redness (positive) and greenness (negative), and (b*) for yellowness (positive) and blueness (negative).

Dye	L*	a*	b*	Average ∆E against blank	H	CV % of K/S	Stdv	K/S	Wavelength (nm)
3a	48.05	5.71	26.53	53.66	91.5	0.408	0.218	22.2	400
3b	33.2	3.87	13.43	62.22	85.9	0.375	0.233	24.3	390
3c	27.21	6.99	7.59	68.17	90.5	0.481	0.328	16.9	390
3d	31.68	8.19	13.52	64.9	88.9	0.475	0.308	18.2	410
4a	42.12	9.91	23.92	56.88	89.8	0.531	0.302	20.5	380
4b	43.7	7.21	25.7	58.42	78.9	0.355	0.207	18.61	400
4c	35.44	12.5	22.67	65.64	83.4	0.391	0.256	14.9	410
4d	37.33	7.51	20.75	62.25	70.2	0.316	0.196	17.53	380

**Table 4:** K/S values and dye fixation for all dyes.

Dyes	(K/S) after dyeing	(K/S) after extraction	Fixation (%)
3a	22.2	21.1	95.04
3b	24.3	23.33	96.01
3c	16.9	15.14	89.58
3d	18.2	17.1	93.95
4a	20.5	19.3	94.14
4b	18.61	17.68	95
4c	14.9	13.55	90.93
4d	17.53	16.2	92.41

### The assessment of antibacterial activity

It was observed how the tinctures (**3a–d)** and (**4a–d)** affected the growth of bacteria like *Escherichia coli, Staphylococcus aureus, Bacillus cereus, and Pseudomonas aeruginosa*. With the aid of diffusion techniques, the antibacterial activity was measured. A sterile cork borer made eight tiny wells that were each 6.3 mm in diameter. The antibacterial activity of the recently developed tinctures (**3a–d)** and (**4a–d)** against four bacterial strains is shown in Table 5. Gram-positive *S. aureus* and *B. cereus*, gram-negative *E. coli*, and *Pseudomonas* are some of these strains. The findings demonstrated that novel diazenyl pyrazole tinctures have superior antibacterial properties to ampicillin. The relevant results for tinctured polyester samples are shown in Table 6, which demonstrates that the samples exhibit exceptional antibacterial activity when compared to blank samples^23^. When compared to other compounds, those containing piperidine and pyrazole moiety displayed stronger antibacterial activity. Indeed, the highest antibacterial activity was exhibited by piperidine moiety containing compound 3b and 3d moreover, the presence of methoxy group in the para position and chlorine atom in the ortho position of benzene linked to the azo functional group in compound 3b, 3c and 3d was associated with increasing in antibacterial activity against staph. *Aureus and B. cereus*, and also in compound 3b, 4a and 4b was associated with increasing in antibacterial activity against *E. coli* and *P. aeruginosa*. The fabric samples exhibiting the highest antibacterial activity were those dyed with 3b and 3d compounds. Importantly, even after five washing cycles, the antibacterial effects of 3b, 3d and 4d dyed fabrics samples remained as its and other compounds was no significant loss in the dyed fabrics.

**Table 5:** Sizes of bacterial colony-clear zones (mm) measured in the vicinity of newly synthesized dyes.

Antibacterial activity (qualitative method) Diameter of the bacterial colony-clear zone (mm)				
Sample	*Escherichia coli* (G −ve)	*Staphylococcus aureus* (G +ve)	*Pseudomonas aeruginosa* (G −ve)	*Bacillus cereus *(G +ve)
3a	6 ± 0.03	8 ± 0.14	9 ± 0.03	6 ± 0.14
3b	18 ± 0.06	21 ± 0.14	18 ± 0.03	22 ± 0.14
3c	12 ± 0.03	25 ± 0.14	14 ± 0.14	20 ± 0.14
3d	14 ± 0.06	20 ± 0.06	15 ± 0.06	19 ± 0.14
4a	15 ± 0.03	14 ± 0.14	11 ± 0.06	9 ± 0.14
4b	15 ± 0.06	13 ± 0.06	16 ± 0.03	18 ± 0.14
4c	13 ± 0.03	12 ± 0.03	9 ± 0.03	6 ± 0.14
4d	12 ± 0.03	12 ± 0.03	15 ± 0.03	10 ± 0.14
Ampicillin	22	18	17	20

**Table 6:** Diameters (mm) of the bacterium colony-clear zones surrounding polyester fabric samples after (once cycle and five cycles) of fabric washing.

Polyester sample (dyed)	The diameter of clear zone (mm)			
	*Bacillus cereus* (G +ve)	*Staphylococcus aureus* (G +ve)	*Pseudomonas aeruginosa* (G −ve)	*Escherichia coli* (G −ve)
**3a**	5 (4)	6 (5)	8 (6)	4 (4)
**3b**	19 (19)	19 (18)	16 (16)	17 (17)
**3c**	17 (15)	22 (20)	11 (9)	10 (8)
**3d**	15 (15)	17 (17)	14 (14)	12 (11)
**4a**	7 (5)	13 (10)	9 (8)	13 (10)
**4b**	17 (15)	9 (6)	14 (12)	12 (8)
**4c**	6 (5)	9 (8)	7 (6)	11 (10)
**4d**	9 (9)	10 (9)	12 (11)	10 (10)

### Raman spectra of the dyed and undyed polyester samples

**Figure 6:**
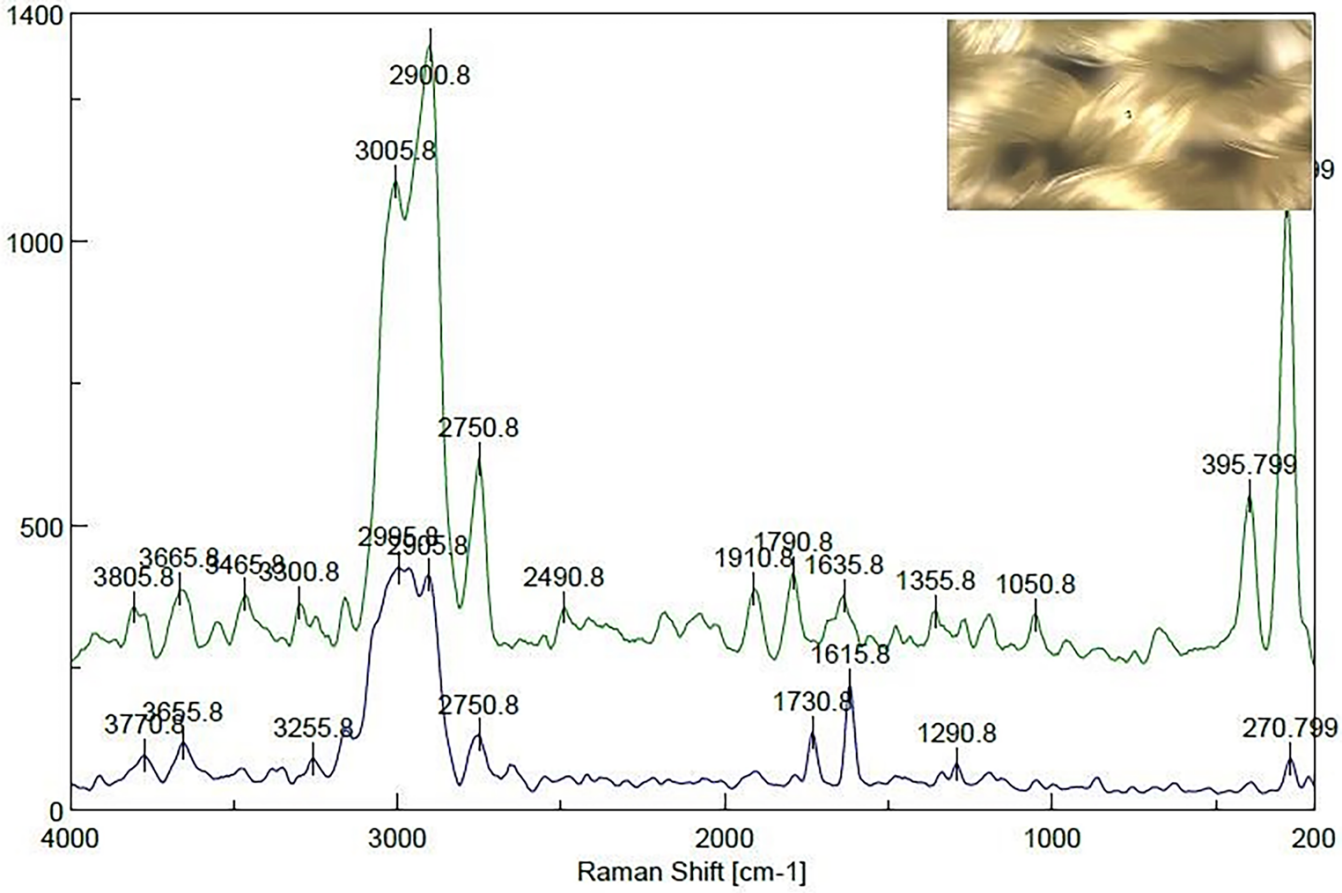
Raman spectra of the undyed and dyed polyester fabrics in scCO_2_. This indicates the dyeing produced effective absorption inside not on the surface of the polyester fabrics.

**Figure 7:**
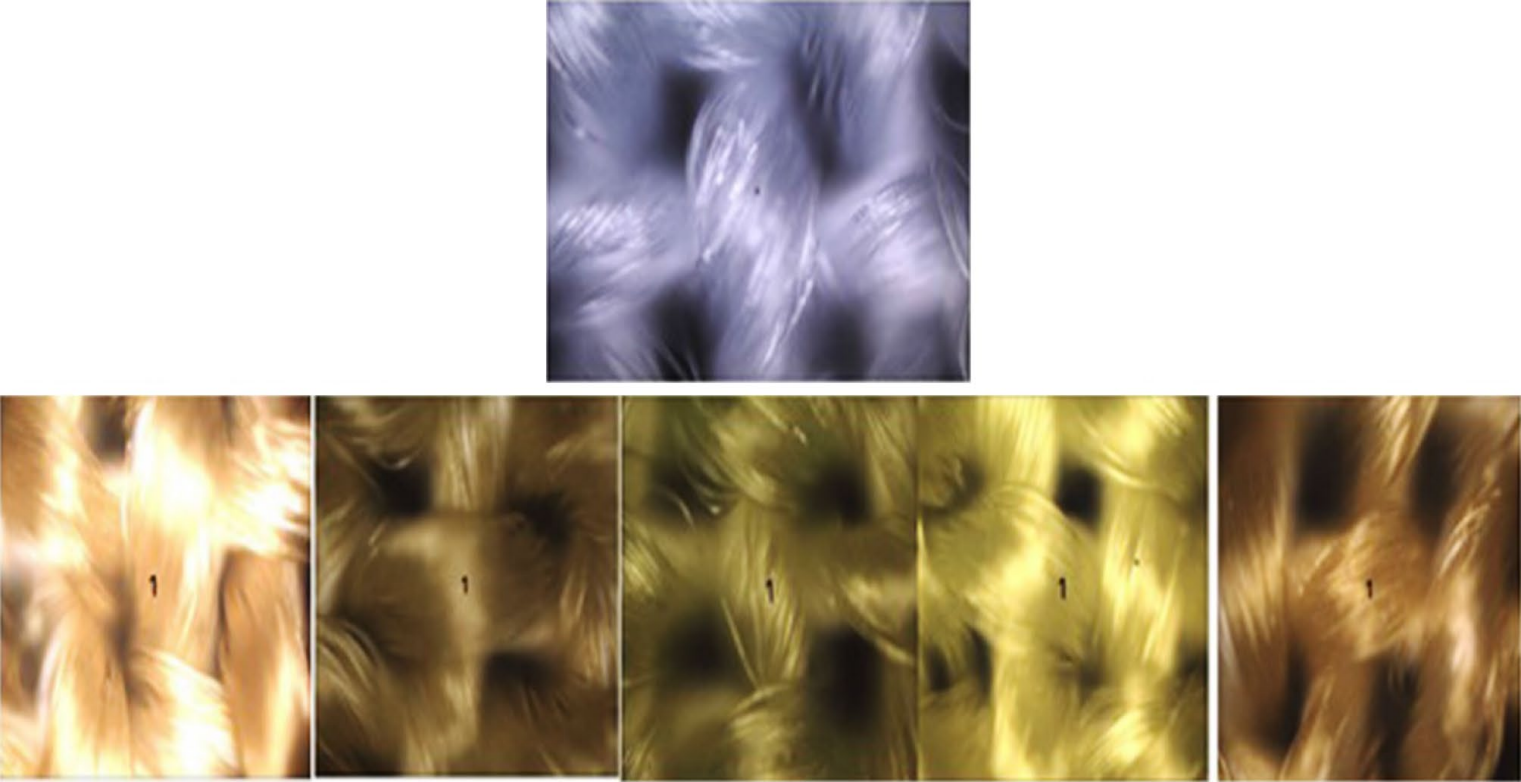
Raman pictures for dyed and undyed polyester fabrics in scCO_2_.

**Figure 8:**
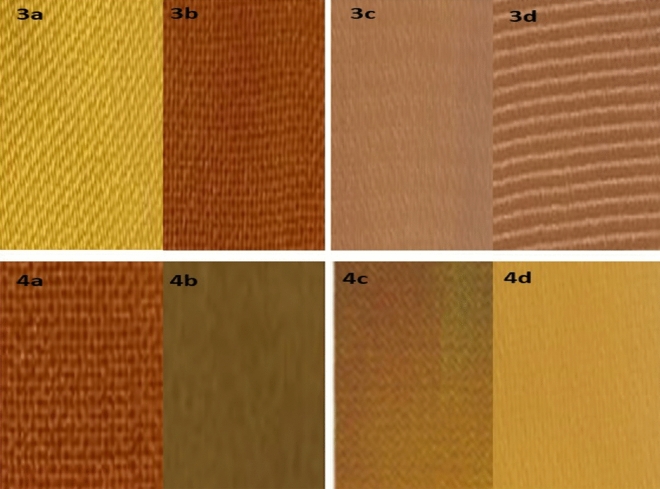
Photograph of the dyed polyester fabrics in scCO_2_ for 3a, 3b, 3c, 3d, 4a, 4b, 4c, and 4d.

Raman analysis was used to characterize the obtained dispersed dyes to assure that the dyeing produced effective absorption inside or on the surface of the polyester fabrics. Figure 6 illustrates a comparison of the Raman spectra for dyed and undyed fabrics. The spectra for fibers analysis were acquired at a wavelength of 200–4000 cm^−1^, scan time settings of (3 s) and a resolution of 2.32 cm^−1^. In Figure 6, the peaks of Raman spectra of the dyed samples of the polyester fabrics were assigned at 3300 (NH), 2900 (CH saturated), 2490 (conjugated CN), 1790 (C=O), and 1635 (C=C aromatic ring) cm^−1^. The absorption bands at 1150–1355 cm^−1^ arise from the characteristic stretching vibrations of the azo groups (–N=N–) in the dye structure. In the Raman spectra of the undyed sample, C=O stretching was observed at cm^−1^, and (CH) saturated were observed at 1730 and 2908.5 cm^−1^, respectively. According to the correctly captured Raman spectra, all the predicted and anticipated distinctive groups were present in the chemical structures of the dyes created. Additionally, the Raman images for dyed and undyed polyester fibers in scCO2 in Figures 7 and 8 validated these observations.

## Conclusions

This work effectively synthesized and dyed polyester fabric using a number of new biologically active dispersion dyes for supercritical carbon dioxide dyeing. 1-((aryl diazenyl) cyanoacetyl) piperidine (**3a–d)** were produced by combining 1-cyanoacetyl piperidine **(1)** with the diazonium salt of aryl amine derivatives (**2a–d)**. When the propane nitriles (**3a–d)** were condensed with hydrazine in an attempt to synthesize the derivatives of the 4-(phenyldiazenyl)-5-(piperidin-1-yl)-1*H*-pyrazol-3-amine dye **(5a)**, the unexpected 3-aminopyrazol-5-ol yellow–red-hued dispersion dyes (**4a–d)** were recognized as the reaction products. The dyeing procedures' outcomes appeared that the highest color strength and fastness (against rubbing, washing, light, and perspiration) were accomplished at 25 MPa, 130 °C, 120 min., and 3% dye concentration for all tincture and 2% dye concentration for (**3d)** dye. The dyed samples gave outstanding coloring uniformity on polyester fabrics, and the color fastness properties ranged from very good to excellent. The dyed fabrics exhibited good potential for antibacterial functionality in dyes 3(a–d) than 4(a–d) due to the presence of piperidine ring. Additionally, Pseudomonas aeruginosa, Escherichia coli, Bacillus cereus, and Staphylococcus aureus were all effectively inhibited by the dyed polyester fabric. The compound 3b dyed PET fabric samples exhibited superior antibacterial activity to all dyes against P. aeruginosa, E. coli, B. cereus, and Staph. aureus. For 3b, 3d and 4d dyed PET fabric samples, much of the antibacterial activity was retained even after five cycles of washing (durability test). Polyester fabrics will soon be industrially dyed with dyes using the energy and environmentally friendly scCO_2_ medium.

### Supplementary Information

Supplementary Information.

## Data Availability

All data generated or analysed during this study are included in this published article [and its supplementary information files].
