# Supplementary material for: Synthesis of novel azo pyrazole disperse dyes for dyeing and antibacterial finishing of PET fabric under supercritical carbon dioxide
Source: Sci Rep. 2024 Jan 11;14:1121. doi: 10.1038/s41598-023-48740-y (PMC10784459; doi:10.1038/s41598-023-48740-y)

**Synthesis of Novel Azo pyrazole Disperse Dyes for Dyeing and antibacterial finishing of PET Fabric under Supercritical carbon dioxide.**

Mamdouh Sofan^1, *^, Fathy El-Taweel^1^, Adel Abdel-Rahman^2^, Hager Salman ^1^ and Elham Negm^1^

**^1^** Chemistry Department, Faculty of Science, Damietta University,

New Damietta, Egypt

^2^ Chemistry Department, Faculty of Science, Menoufia University,

Shebin El-Koam, Egypt

^*^corresponding. [masofan1953@du.edu.eg](mailto:masofan1953@du.edu.eg).

# Compound 3a

# IR-Spectra:


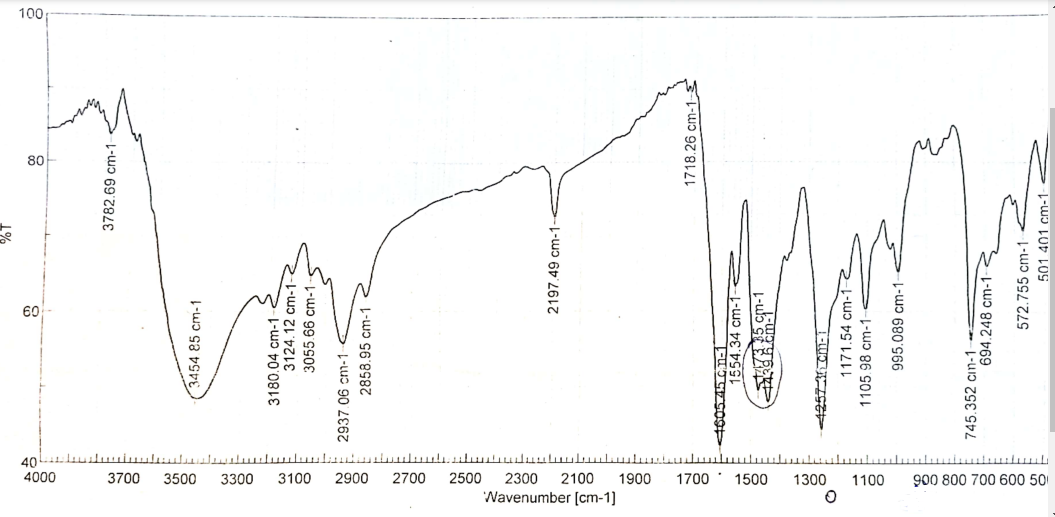


# ^1^H-NMR data:


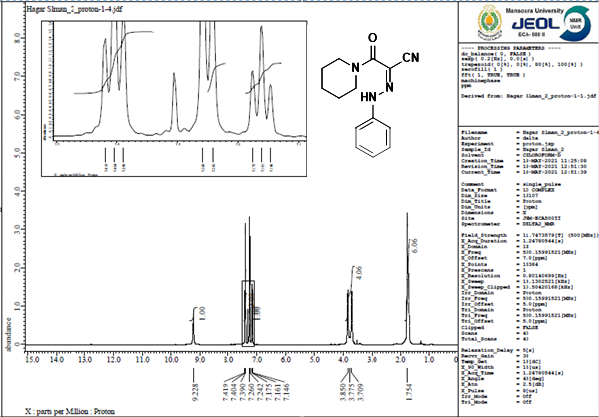


# ^1^H-NMR data (D_2_O):


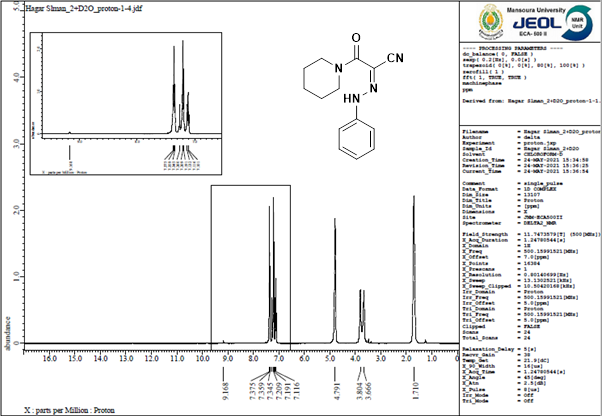


# Mass spectra


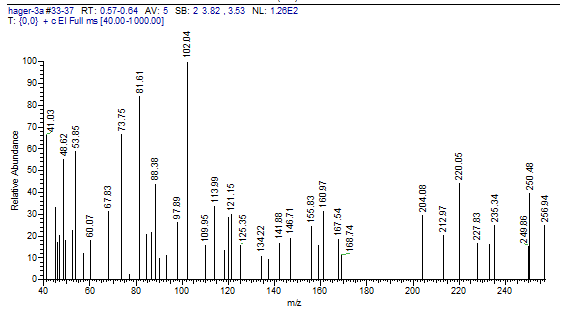


# Compound 3b:

# IR-Spectra:


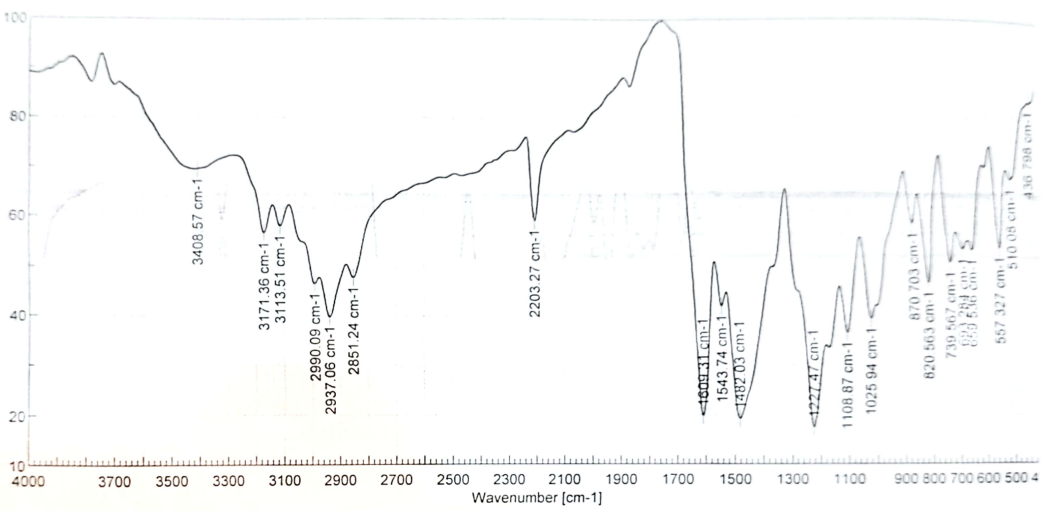


# ^1^H-NMR data:


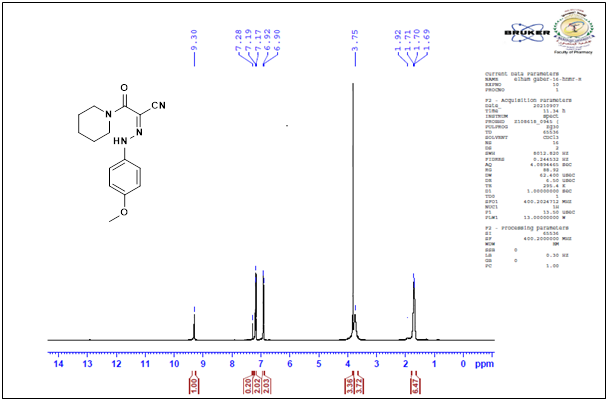


# ^13^C-NMR data:

#
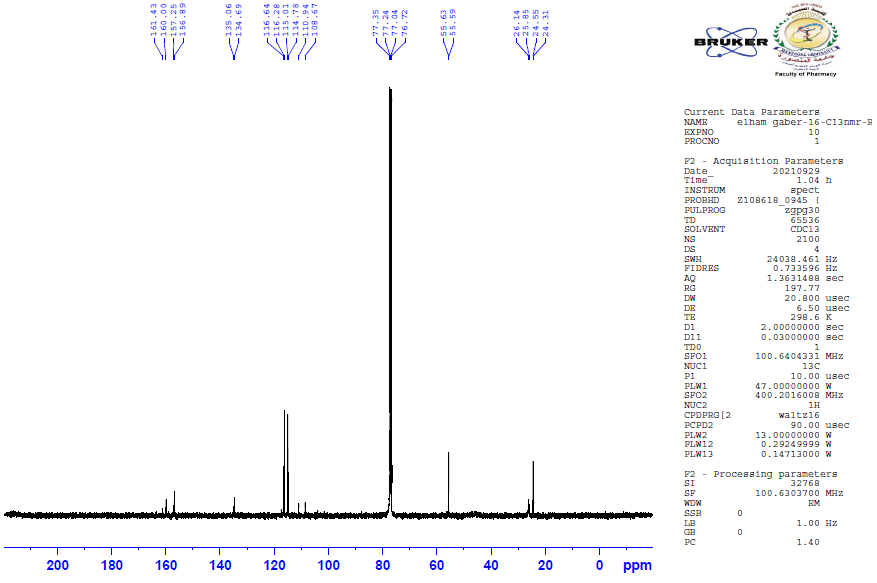

# Mass spectra


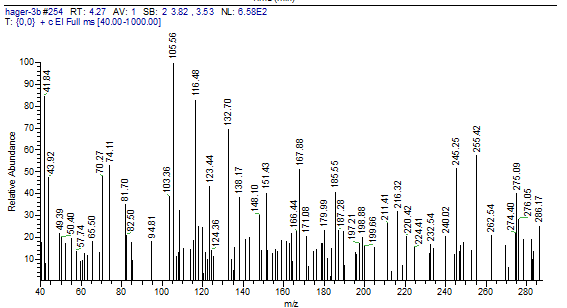


# Compound 3c

# IR-Spectra:


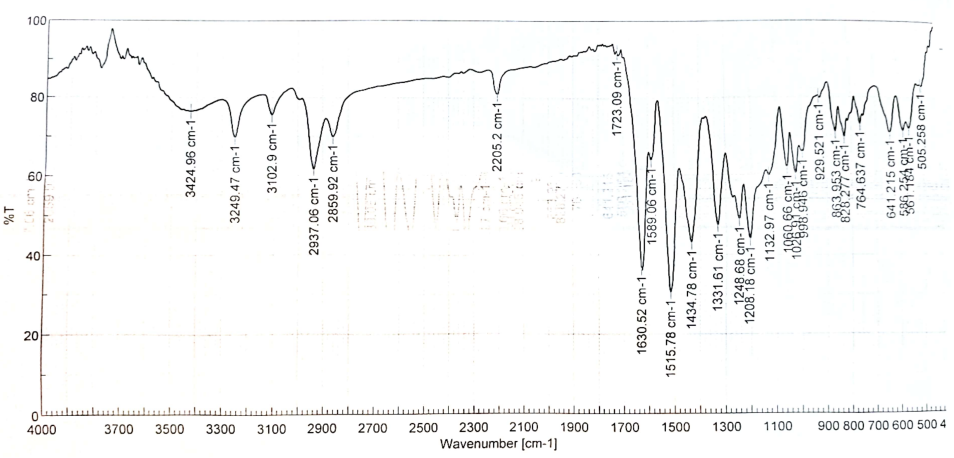


# ^1^H NMR data:


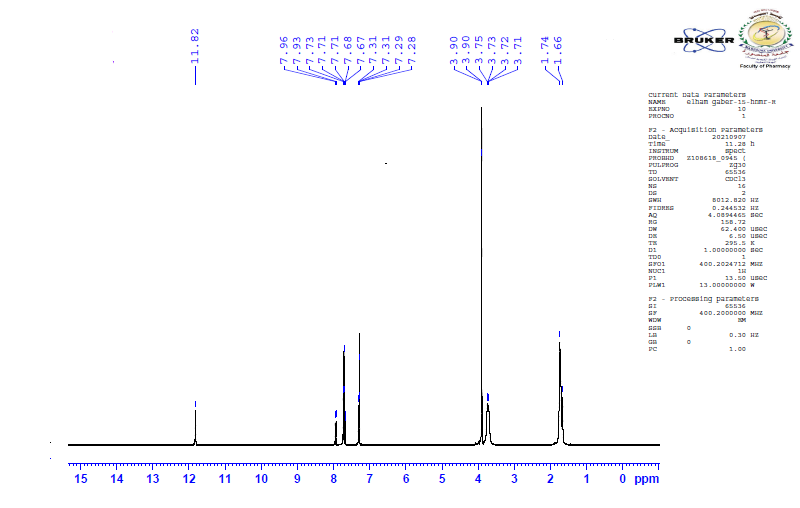

# ^13^C-NMR data:


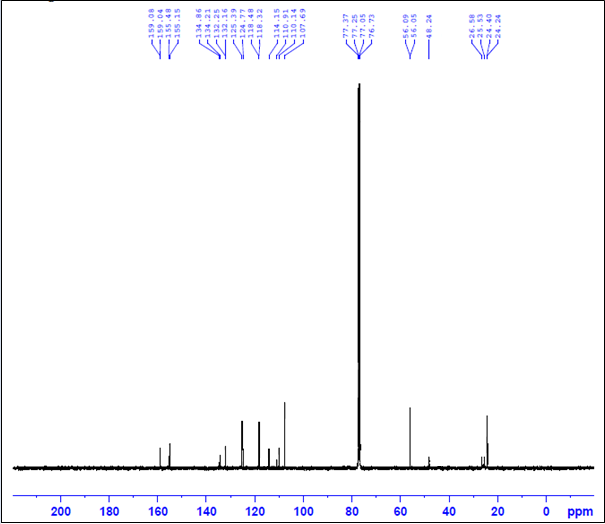

# Compound 3d

# IR-Spectra:


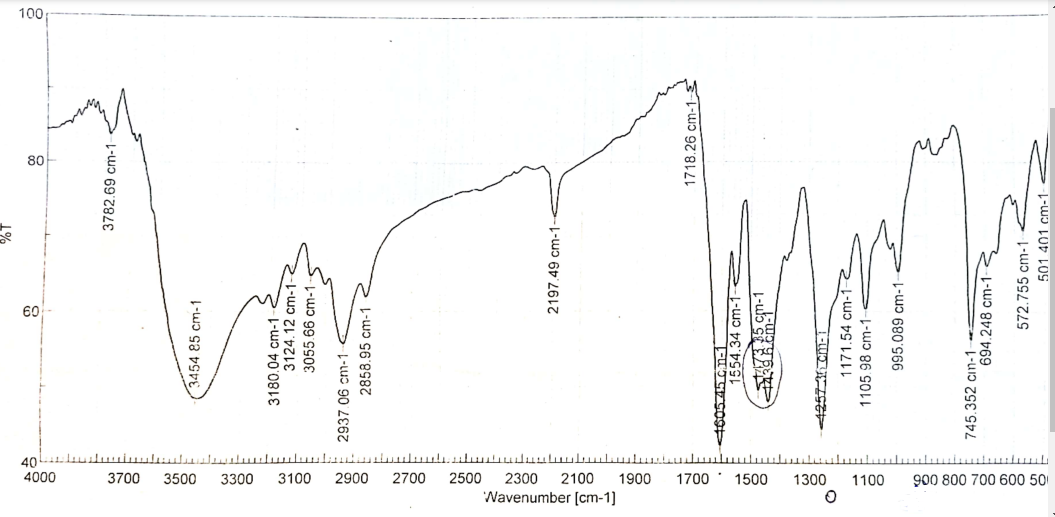


# ^1^H NMR data:


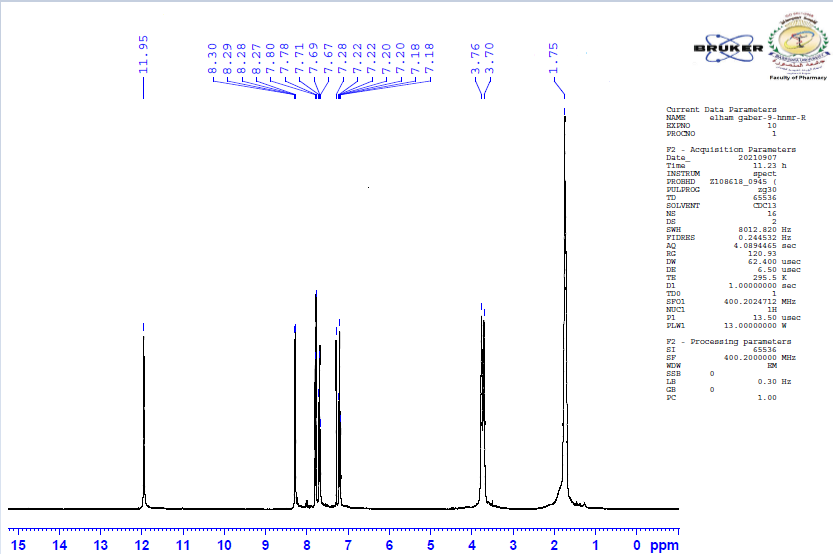

# ^13^C-NMR data:


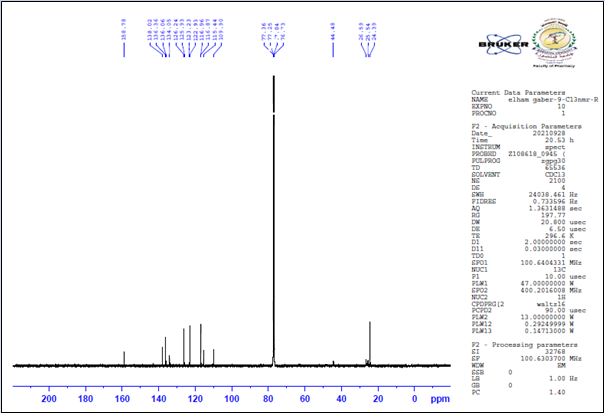

# Compound 4a

# IR-Spectra:


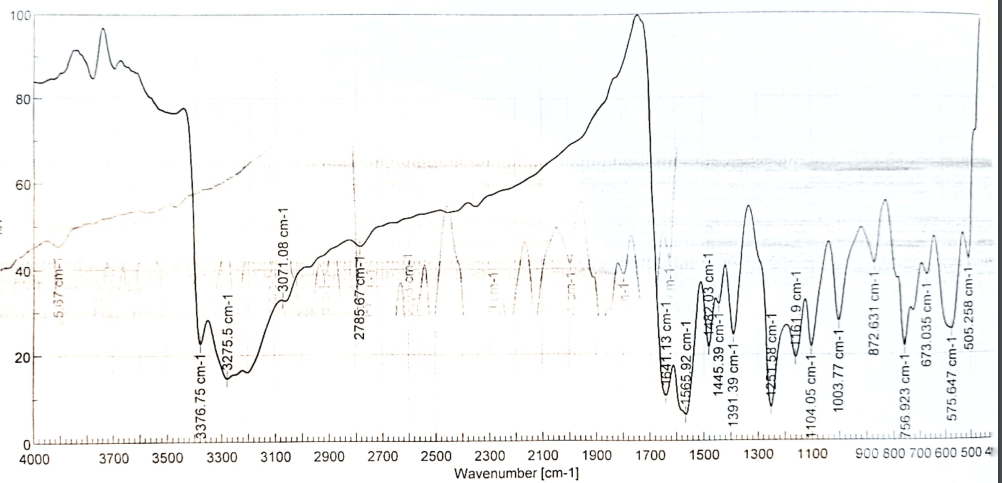


# ^1^H NMR data:


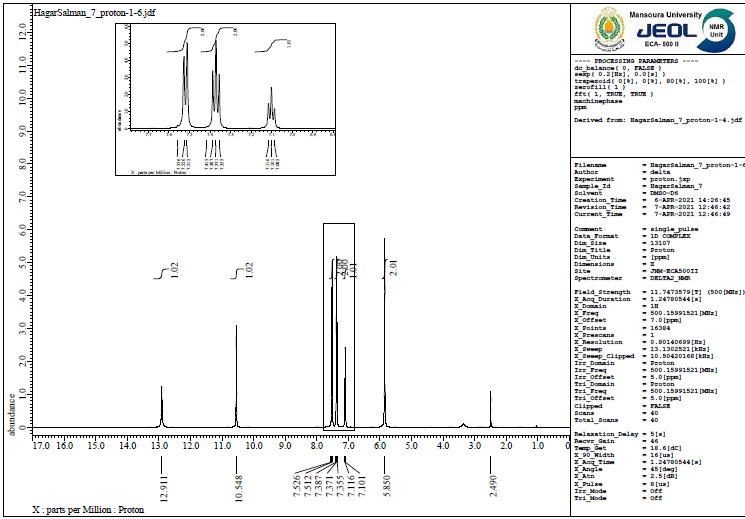

# ^1^H-NMR data (D_2_O):


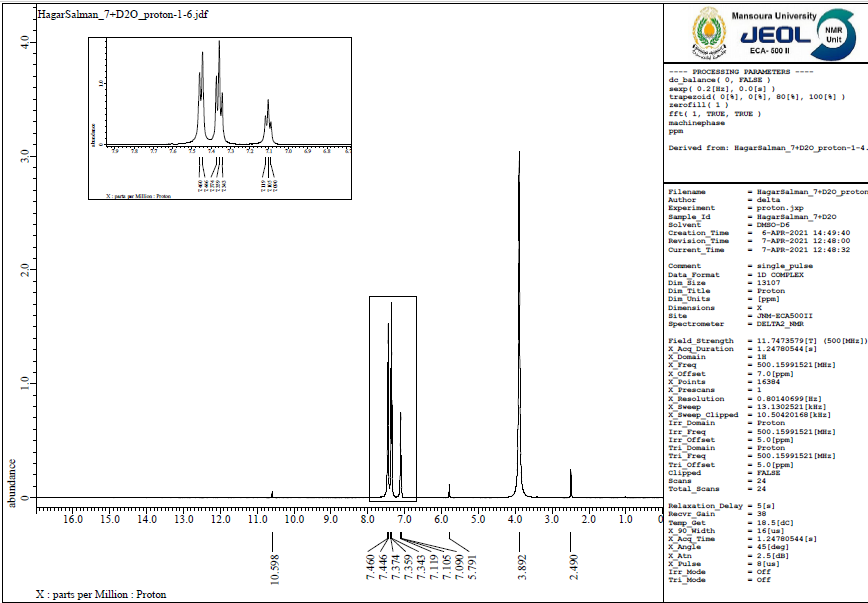


# Mass spectra:


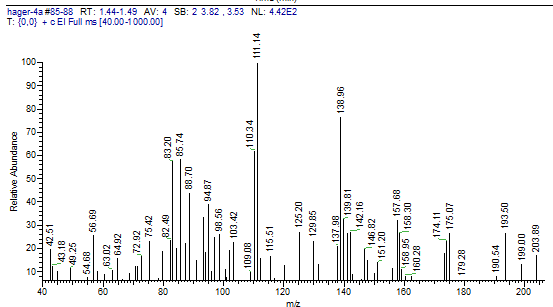


# Compound 4b

# IR-Spectra:


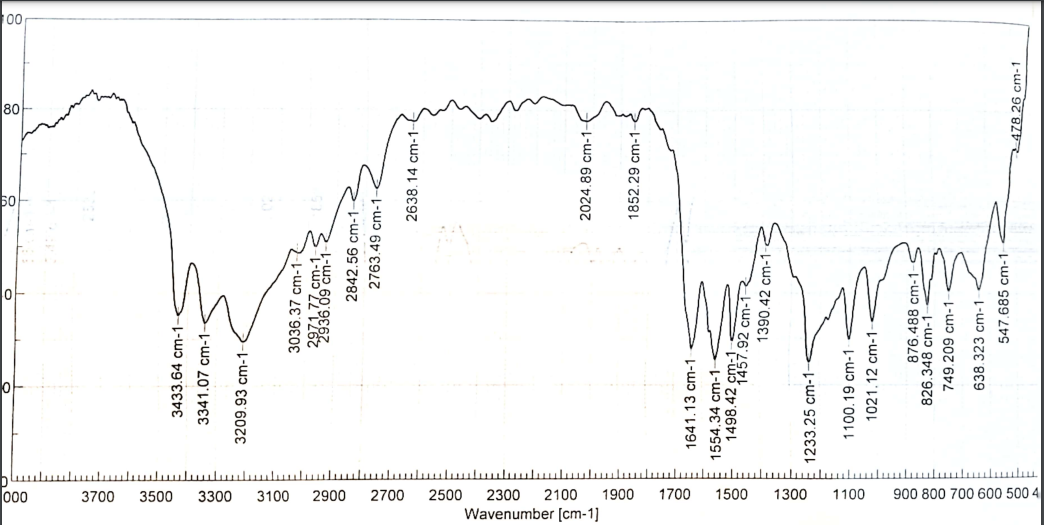


# ^1^H NMR data:


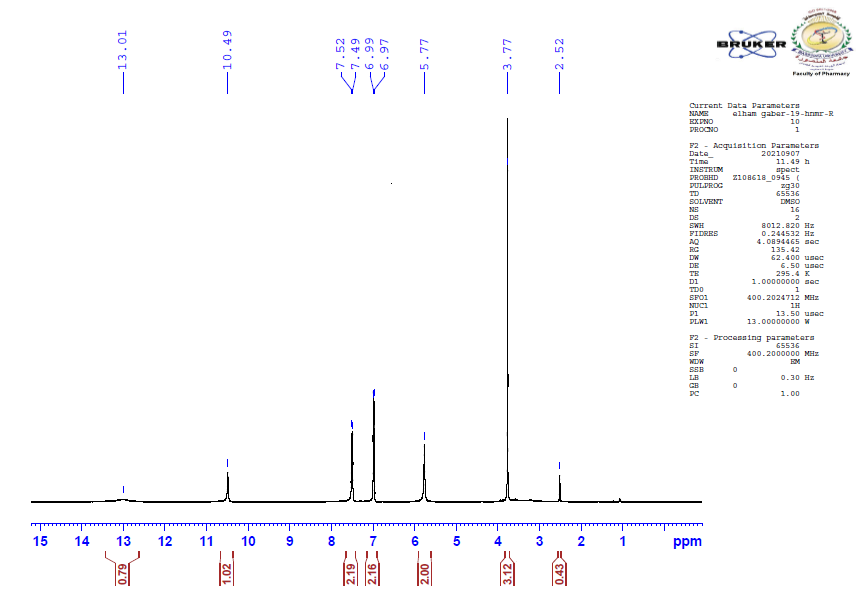

# ^1^H-NMR data (D_2_O):


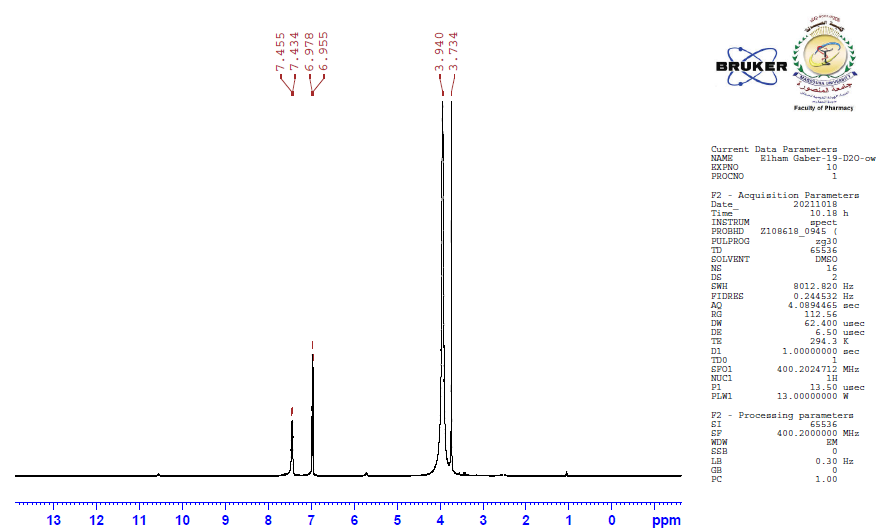

# ^13^C-NMR data:


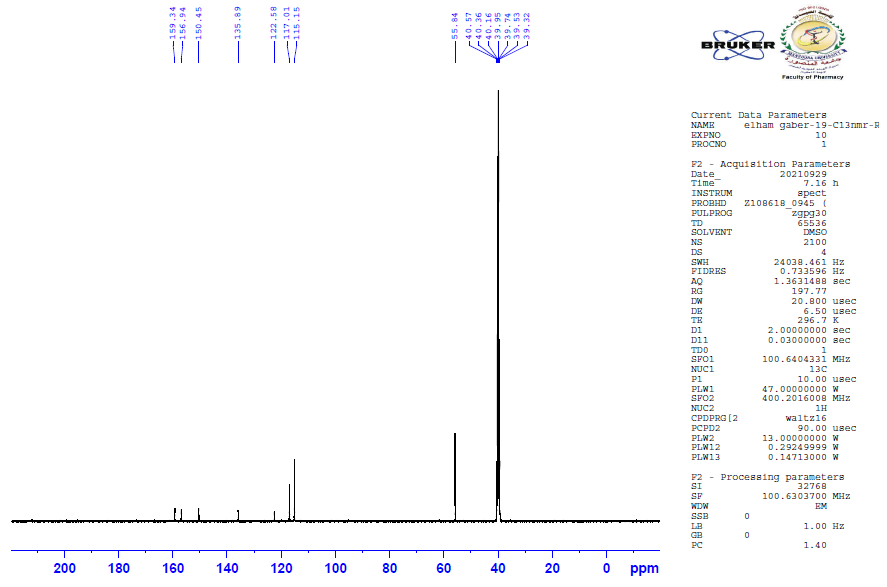

# Mass spectra:


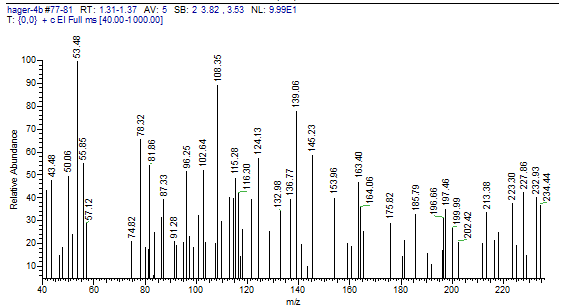


# Compound 4c

# IR-Spectra:


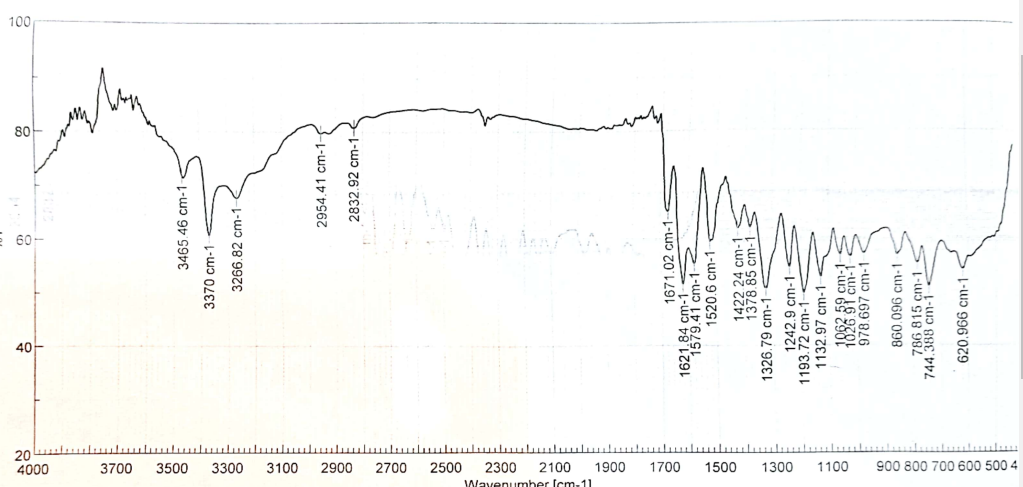


# ^1^H NMR data:


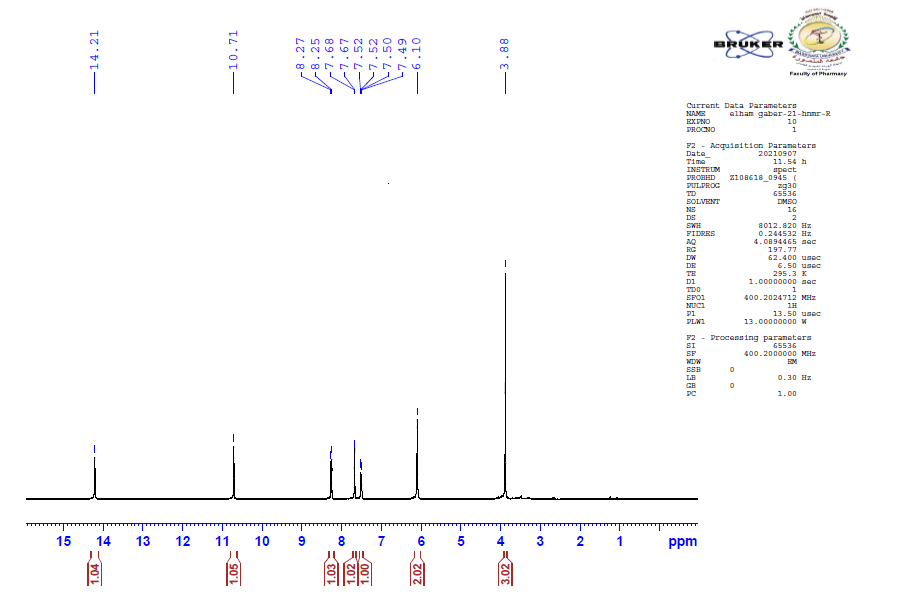

# ^1^H-NMR data (D_2_O):


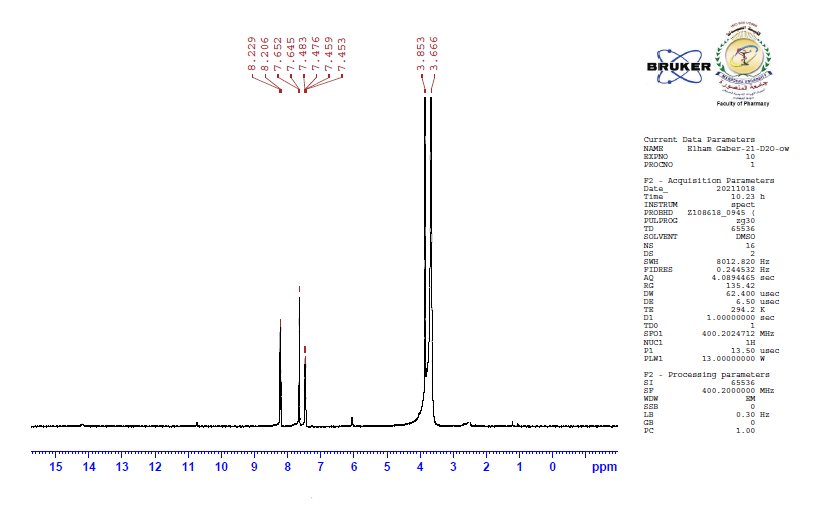

# ^13^C-NMR data:


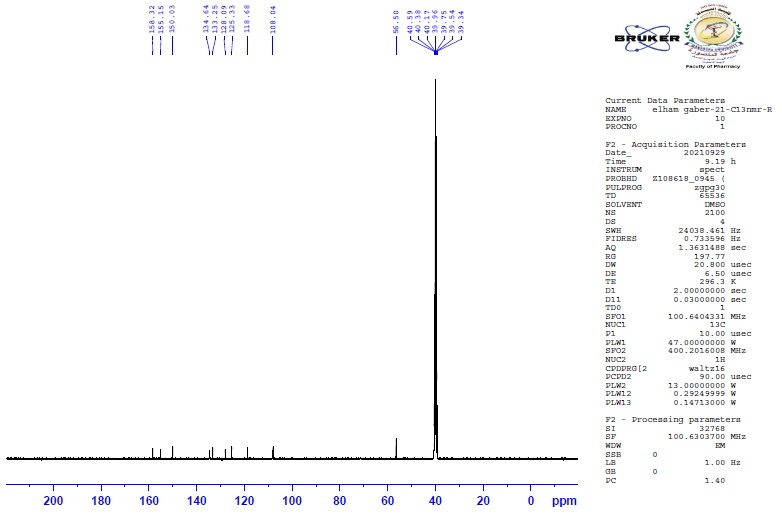

# Compound 4d

# IR-Spectra:
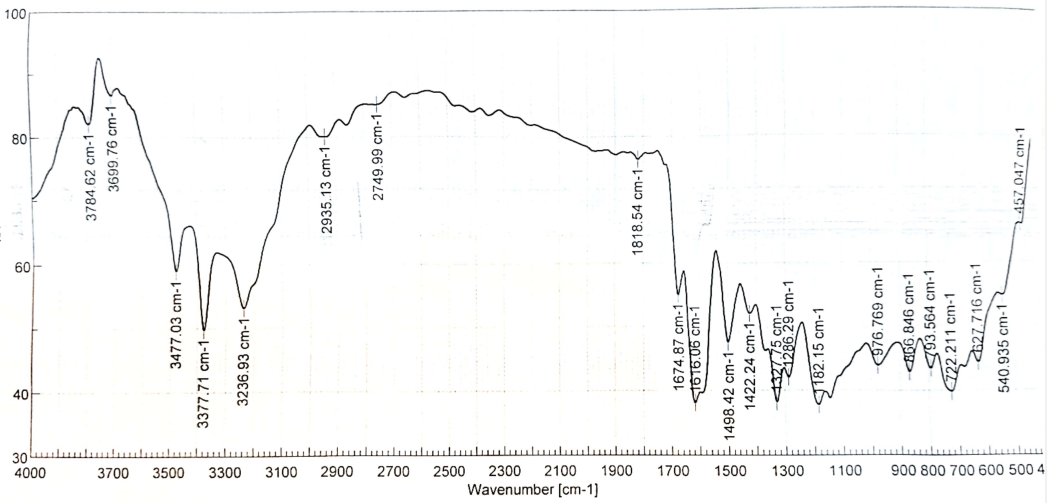


# ^1^H-NMR data:


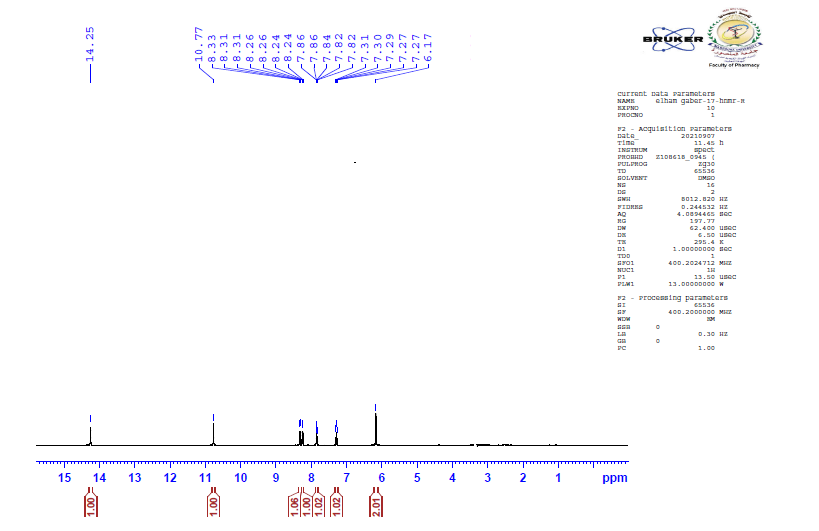

# ^1^H-NMR data (D_2_O):


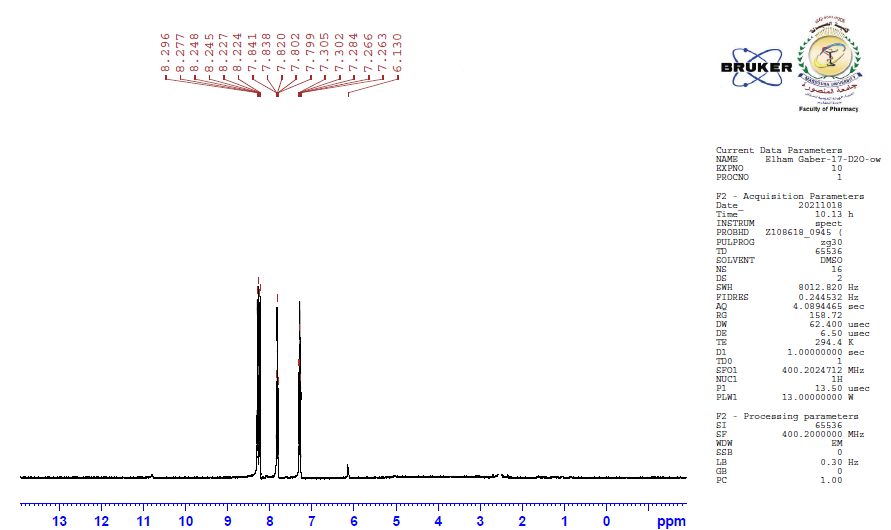

# ^13^C-NMR data:


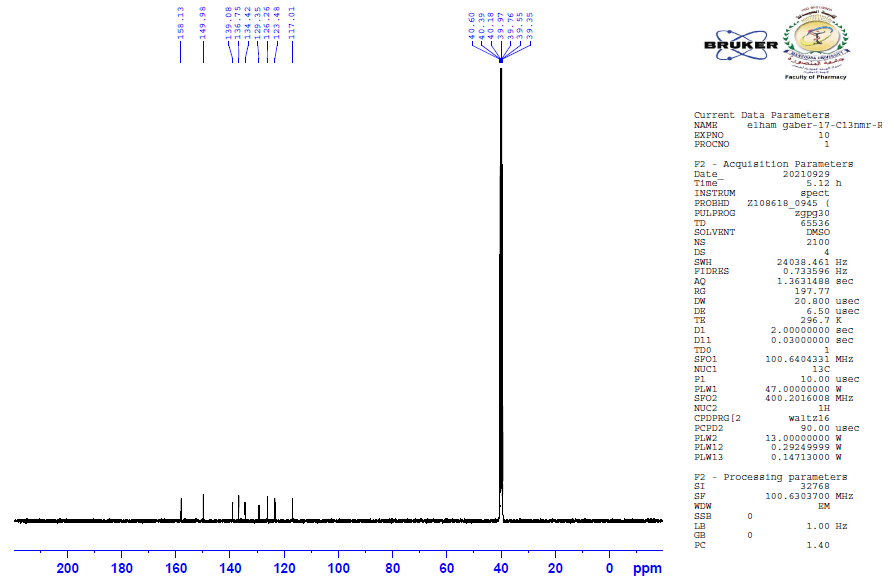

Supplement: Supplementary file 1 — Supplementary Information. [file 41598_2023_48740_MOESM1_ESM.docx]
